# The role of the mucosal barrier system in maintaining gut symbiosis to prevent intestinal inflammation

**DOI:** 10.1007/s00281-024-01026-5

**Published:** 2024-11-26

**Authors:** Ryu Okumura, Kiyoshi Takeda

**Affiliations:** 1 Department of Microbiology and Immunology, Graduate School of Medicine, Osaka University, Suita, Osaka 565-0871 Japan; 2 WPI Immunology Frontier Research Center, Osaka University, Suita, Osaka 565-0871 Japan; 3 Institute for Open and Transdisciplinary Research Initiative, Osaka University, Suita, Osaka 565-0871 Japan; 4 Center for Infectious Disease Education and Research, Osaka University, Suita, Osaka 565-0871 Japan

**Keywords:** Mucus, Glycosylation, Antimicrobial peptide, Inflammatory bowel diseases

## Abstract

In the intestinal tract, where numerous intestinal bacteria reside, intestinal epithelial cells produce and release various antimicrobial molecules that form a complex barrier on the mucosal surface. These barrier molecules can be classified into two groups based on their functions: those that exhibit bactericidal activity through chemical reactions, such as antimicrobial peptides, and those that physically hinder bacterial invasion, like mucins, which lack bactericidal properties. In the small intestine, where Paneth cells specialize in producing antimicrobial peptides, the chemical barrier molecules primarily inhibit bacterial growth. In contrast, in the large intestine, where Paneth cells are absent, allowing bacterial growth, the primary defense mechanism is the physical barrier, mainly composed of mucus, which controls bacterial movement and prevents their invasion of intestinal tissues. The expression of these barrier molecules is regulated by metabolites produced by bacteria in the intestinal lumen and cytokines produced by immune cells in the lamina propria. This regulation establishes a defense mechanism that adapts to changes in the intestinal environment, such as alterations in gut microbial composition and the presence of pathogenic bacterial infections. Consequently, when the integrity of the gut mucosal barrier is compromised, commensal bacteria and pathogenic microorganisms from outside the body can invade intestinal tissues, leading to conditions such as intestinal inflammation, as observed in cases of inflammatory bowel disease.

## Introduction

The intestine is a distinct organ where heterogeneous organisms, including bacteria, fungi and viruses, coexist alongside host cells, establishing mutual relationships between commensal microorganisms and the host. Given that these symbiotic organisms can potentially become targets for host immune cells, segregation between symbionts in the lumen and immune cells in the lamina propria is required to maintain a balanced environment in the gut. Intestinal epithelial cells, lining the mucosal surface, play a crucial role in achieving this segregation. They not only absorb nutrients and water but also contribute to this separation by releasing antimicrobial molecules and forming a mucus layer. Collectively, these components are referred to as mucosal barriers. The expression of these mucosal barriers is altered in response to bacterial signals from the lumen and cytokines from the lamina propria to enable adaptation to alterations in the gut environment, including changes in intestinal bacterial populations and the presence of pathogenic bacterial infections. Therefore, when this well-organized barrier system is genetically compromised or disrupted by certain environmental factors, intestinal bacteria can invade intestinal tissue, resulting in intestinal inflammation, as observed in inflammatory bowel disease (IBD).

In this review, we discuss the intestinal barrier system, classifying barrier molecules into chemical and physical components. We also review the mechanisms that regulate the expression of mucosal barrier molecules by intestinal bacteria and lamina propria cells, including immune cells. Finally, we discuss recent advances in understanding the mechanisms underlying intestinal inflammation in cases of mucosal barrier dysfunction and future research prospects on gut mucosal barriers.

## Intestinal epithelial barrier and control of intestinal *bacteria*

**Fig. 1:**
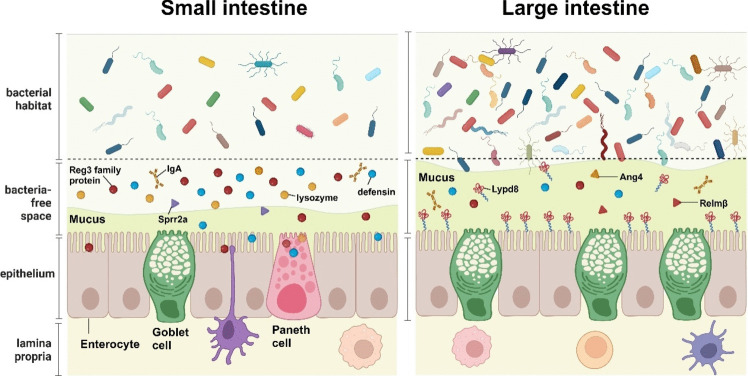
Structure of the mucosal barrier in the small and large intestines. In the small intestine, chemical barrier molecules, including defensins, Reg3 family proteins, Lysozyme, and small proline-rich protein 2a (Sprr2a), are primarily produced by Paneth cells. These components play a crucial role in maintaining a separation between intestinal bacteria and intestinal epithelial cells in the small intestine by killing bacteria coming close to intestinal epithelia. In contrast, in the large intestine, where Paneth cells are absent, chemical barrier molecules including angiogenin, ribonuclease A family member 4 (Ang4), and resistin-like molecule β (Relmβ) are not as prominent. Instead, the large intestine relies on the constitutively high expression of mucin 2 (Muc2) by goblet cells and LY6/PLAUR domain-containing 8 (Lypd8) by enterocytes, both of which are extensively glycosylated proteins. The thick mucus layer, composed of polymerized Muc2, forms a network structure that effectively segregates a vast population of intestinal bacteria from direct contact with intestinal epithelial cells. Lypd8 plays a critical role in promoting this segregation by inhibiting bacterial invasion, particularly in the case of flagellated bacteria. Overall, the small and large intestines employ distinct strategies for maintaining the mucosal barrier, with the small intestine relying on Paneth cell-secreted chemical defenses and the large intestine utilizing glycosylated proteins like Muc2 and Lypd8 to establish an effective barrier against bacterial contact

Unlike other organs, the small intestine and large intestine harbor diverse bacterial species that the immune system would typically recognize as foreign. To prevent direct confrontation between immune cells in the lamina propria and gut bacteria residing in the lumen, intestinal epithelial cells themselves exist as a barrier, and express a variety of barrier molecules, which effectively segregate the intestinal bacteria from the intestinal tissue. In this context, we will discuss the mucosal barrier system, which consists of epithelial cell-derived chemical and physical barrier molecules and plasma cell-derived immunoglobulin A (IgA), and its role in controlling intestinal bacteria in the small and large intestines (Fig. 1).

## Chemical barrier molecules

The secreted chemical barrier molecules induce chemical changes in microorganisms, encompassing a group of molecules with antimicrobial activity. These various bactericidal molecules are abundantly produced, mainly in the small intestinal epithelial cells including Paneth cells (Table 1). One class of representative molecules secreted are antimicrobial peptides (AMPs), which are primarily produced by Paneth cells. AMPs are short peptides of approximately 20 to 50 amino acids in length that are predominantly composed of basic amino acids, e.g., as arginine and lysine [1, 2]. These peptides are conserved across a wide range of organisms, from insects and plants to higher vertebrates. AMPs possess a positively charged basic region and a hydrophobic region. The positively charged region of an AMP binds to the negatively charged bacterial membrane, whereas the hydrophobic region alters the permeability of the bacterial membrane, thereby exerting its killing activity [3, 4]. Among AMPs, defensin is a representative AMP, consisting of three family members (α, β and θ-defensin) [5]. Among them, α-defensin, known as cryptdin, is primarily and abundantly produced by Paneth cells in the small intestine, while β-defensin is expressed at low levels in the small and large intestinal epithelial cells and θ-defensin is found only in rhesus monkeys. α-defensin plays a pivotal role in the innate immunity of the small intestine by cooperating with cathelicidin, which is produced by intestinal epithelial cells including enterocytes, goblet cells and Paneth cells [6, 7]. Pro-cryptdin is converted into mature cryptdin by matrix metalloproteinase-7 (MMP-7). MMP-7 deficiency results in a high susceptibility to *Salmonella* Typhimurium infection because mature cryptdin is absent [4]. Human cathelicidin consists of 37 amino acids, with two leucines at the N-terminus, and is hence referred to as LL-37 [8]. In addition to the properties of AMPs, LL-37 is known to strongly neutralize endotoxins, providing a defense mechanism against endotoxin shock [9, 10].

**Table 1:** Antimicrobial molecules expressed in intestinal epithelial cells

Antimicrobial molecule	Tissue	Call type
α-defensin	small intestine	Paneth cell
β-defensin	small and large intestine	Paneth cell, enterocyle
Cathelicidin (LL-37)	small intestine	Paneth cell, enterocyle
Lysozyme	small intestine	Paneth cell
Phospholipase A2	small and large intestine	Paneth cell, enterocyle
Regenerating islet-derived protein 3γ	small > large intestine	Paneth cell, enterocyle
Resistin-like molecule β	large intestine	goblet cell, enterocyle
Small proline-rich protein 2a	small and large intestine	enterocyle
Angiogenin 4	small and large intestine	Paneth cell, goblet cell

The table presents antimicrobial molecules expressed by intestinal epithelial cells, along with the tissues and cell types that express each molecule, based on previous reports referenced in this paper and publicly available RNA-sequencing data

The regenerating islet-derived protein 3 (Reg3) family of proteins are C-type lectins that bind to the bacterial cell surface and exert antimicrobial activity by forming pores, similar to defensins [11, 12]. Reg3β and Reg3γ are produced abundantly by intestinal epithelial cells, including Paneth cells in the small intestine. Reg3β reportedly binds directly to lipopolysaccharides (LPS) and exerts bactericidal activity against Gram-negative bacteria [13]. In contrast, Reg3γ exhibits a killing activity on Gram-positive bacteria by binding to their surface peptidoglycans, followed by forming a membrane-penetrating pore [12, 14, 15]. Particularly, Reg3γ mainly contributes to the spatial separation of intestinal bacteria and the small intestinal epithelia through inhibiting the invasion of mucosal surface by Gram-positive bacteria, such as *Eubacterium rectale* and segmented filamentous bacteria (SFB) [15].

Lysozyme, another hydrolytic enzyme produced by Paneth cells, exhibits bactericidal antimicrobial activity against Gram-positive bacteria by breaking the bond between N-acetylmuramic acid and N-acetylglucosamine, which are the main components of the bacterial cell wall peptidoglycan [16]. Furthermore, phospholipase A2 (PLA2) is an enzyme that hydrolyzes the fatty acid ester bond of phospholipids and exerts antimicrobial activity by hydrolyzing the cell membrane of Gram-positive bacteria [17, 18]. Resistin-like molecule β (Relmβ) was recently reported to be a bactericidal protein that binds to negatively charged lipids and forms a multimeric pore in the membranes of Gram-negative bacteria [19]. Relmβ is abundantly produced by intestinal epithelial cells in the colon, where it promotes the segregation of gut bacteria and the epithelia [19] [20]. Small proline-rich protein 2a (Sprr2a) was also reported to possess killing activity against Gram-positive bacteria such as *Listeria monocytogenes* by permeabilizing membranes [21]. Interestingly, Sprr2a expression is induced by Th2 cytokines during helminth infection and protects against helminth-induced bacterial invasion of intestinal tissue [21]. Angiogenin 4 (Ang4), harboring ribonuclease activity and abundantly expressed in the small and large intestines, exhibits microbicidal activity against bacterial and fungal pathogens by disrupting bacterial membrane integrity [22, 23]. These antimicrobial agents prevent the invasion by intestinal bacteria of the intestinal tissues effectively, particularly in the small intestine, and help avoid excessive immune responses by immune cells against intestinal bacteria. Paneth cells play a critical role in maintaining the barrier integrity not only by producing these chemical barrier molecules, but also by regulating angiogenesis stem cell proliferation, contributing to epithelial cell renewal and wound healing [24, 25].

## Physical barrier molecules

Unlike the chemical barrier, the physical barrier refers to a group of molecules and structures that act as physical defenses to prevent bacterial invasion without directly killing bacteria. In contrast to the small intestine, where there are fewer bacteria due to the higher oxygen level than the large intestine and the presence of Paneth cells, the large intestine, having a significantly greater number of bacteria, primarily relies on the physical barrier to prevent bacterial invasion of the intestinal tissue. The physical barrier includes the mucus layer covering the intestinal mucosa, the glycoprotein cluster known as glycocalyx on the surface of intestinal epithelial cells and the cell–cell adhesion apparatus that holds the intestinal epithelial cells together.

The mucus layer is a structural entity formed by the polymerization of mucin, a highly glycosylated protein produced by goblet cells. In the intestine, Mucin 2 (Muc2), the primary secreted gel-forming mucin, forms a network structure via N-terminal trimerization and C-terminal dimerization through disulfide bonds [26, 27]. Numerous *O*-linked glycans attached to the proline/threonine/serine-rich (PTS) domain of Muc2 contribute to the viscosity of mucus and also bind competitively to pathogenic microorganisms attempting to adhere to the surface of epithelial cells, thereby preventing bacterial invasion and adhesion [28]. In the large intestine, where a vast number of bacteria reside, a substantial amount of mucus is produced by a greater number of goblet cells compared with the small intestine. This greater production generates a thick mucus layer covering the intestinal epithelium, which helps to maintain the mucosal surface in an almost sterile state. With regard to the barrier system of mucus in the large intestine, Hansson and colleagues have elegantly demonstrated a dual-layered system. In this system, an inner-dense layer that attaches to epithelial cells acts as a repellent barrier, whereas an outer-loose layer facilitates microbial interactions [29]. However, notably, this model has limitations and does not consider the effects of peristalsis and material passage on mucus dynamics. Thus, Xia et al. recently proposed a new “encapsulation” model [30]. According to this model, detached mucus from the proximal colon wraps around gut microbiota, dividing microbiota communities into discrete units within fecal pellets. Subsequently, the mucus-encapsulated fecal pellet moves and is further covered with distal colon-derived mucus that has different glycosylation from the proximal colon [30]. Further studies using live imaging are required to confirm the validity of either model.

Glycans play a pivotal role in the mucus barrier function within the gastrointestinal tract, as demonstrated by several previous studies involving various glycosyltransferase-mutant mice [28, 30–34]. For example, mice deficient in core 1 synthase, specifically glycoprotein-N-acetylgalactosamine 3-beta-galactosyltransferase 1 (C1galt1), a crucial enzyme in the biosynthesis of core 1 or 2 *O*-glycans, exhibited a defective mucus layer in the colon, leading to severe colitis resembling that seen in Muc2-deficient mice [32]. In contrast, B3gnt6-deficient mice, which lack the core 3 *O*-glycan, did not develop spontaneous colitis but displayed heightened susceptibility to dextran sulfate sodium (DSS)-induced experimental colitis because of a weakened mucus barrier [31]. These findings underscore the essential role of *O*-glycans in mucus barrier function. However, the specific glycan structure responsible for maintaining the mucus barrier remains unclear. In this context, several recent studies investigated the gene expression profile of glycogenes, including glycosyltransferase and sulfotransferase genes, in intestinal epithelial cells from different sites within the intestine [33–35]. A recent study showed that ST6 (alpha-N-acetyl-neuraminyl-2,3-beta-galactosyl-1,3)-N-acetylgalactosaminide alpha-2,6-sialyltransferase 1 (St6galnac1) is highly expressed in colonic goblet cells, and sialylation of *N*-glycans of Muc2 mediated by St6galnac1 is required for the protection against excessive bacterial proteolytic degradation of Muc2 [33]. Another study demonstrated that disialylated glycans of *O*-glycans of Muc2 synthesized through the actions of UDP-Gal:betaGlcNAc beta 1,3-galactosyltransferase, polypeptide 5 (B3galt5) and ST6 (alpha-N-acetyl-neuraminyl-2,3-beta-galactosyl-1,3)-N-acetylgalactosaminide alpha-2,6-sialyltransferase 6 (St6galnac6), both are highly and specifically expressed in the colon, contribute to the maintenance of mucus mechanical properties and the multidirectional mucin network, inhibiting bacterial invasion of the intestinal tissue [34]. These findings suggest that α2-6 sialic acid imparts a negative charge and hydrophilicity to Muc2, leading to the uniformization of polymerized mucins in a water-reduced environment in the distal colon, which is required for the protection against bacterial invasion and proteolytic degradation of mucins.

The mucus layer contributes predominantly to segregating gut bacteria from intestinal epithelia, whereas other types of barrier molecules are required to maintain this segregation. LY6/PLAUR domain-containing 8 (Lypd8), highly expressed on the surface of intestinal epithelial cells, has been identified to inhibit bacterial invasion within the mucus layer [36, 37]. Lypd8, a GPI-anchored and highly N-glycosylated protein, is continuously shed from intestinal epithelial cells and secreted into the intestinal lumen. Secreted Lypd8 preferentially binds to flagellated bacteria, inhibiting their invasion of colonic epithelia by suppressing their motility [36, 37]. Additionally, Lypd8 has been reported to inhibit the attachment of pathogenic bacteria, such as *Citrobacter rodentium*, by binding to intimin, a virulence factor essential for *C. rodentium* attachment to epithelial cells [38]. Moreover, a recent study demonstrated that Lypd8 contributes to protecting against gut graft-versus-host disease following allogeneic hematopoietic stem cell transplantation by suppressing bacterial translocation from the colon [39]. These findings underscore the crucial role of Lypd8 in preventing bacterial invasion of colonic tissue.

Although the thick mucus layer containing various antimicrobial molecules protects the large intestinal mucosa, those few invading microbes that manage to penetrate this mucus layer face additional physical barriers that prevent their invasion into the intestinal tissues. One such barrier is the glycocalyx, a cluster of sugar chains present on the microvilli surface of intestinal epithelial cells. The glycocalyx, which forms a meshwork of carbohydrate moieties on glycolipids or glycoproteins, including transmembrane mucins such as MUC1, MUC13 and MUC17 (a mouse homolog of Muc3), constitutes the second line of defense against invading bacteria [40]. These transmembrane mucins shield the gut tissue from enteric bacterial pathogens. For example, mice deficient in MUC1 exhibit a high susceptibility to *Campylobacter jejuni* and *Helicobacter pylori* infections [41, 42]. In the case of *H. pylori* infection, MUC1 cleaved by proteases acts as a decoy that prevents *H. pylori* attachment to epithelial cells [43]. Furthermore, the knockdown of *Muc13* increases the attachment of enterotoxigenic *Escherichia coli* to intestinal epithelial cells, indicating that MUC13 also protects the host against the attachment of pathogenic bacteria [44]. Moreover, reduced levels of MUC17 are associated with increased permeability and enhanced bacterial invasion by enteroinvasive *E. coli* [45]. Glycans are indispensable for the barrier functions of these highly glycosylated barrier proteins, mucins and Lypd8. The densely packed sugar chains are believed to physically impede bacterial invasion into the intestinal tissue. However, it remains unclear which glycans are involved in the barrier functions of the glycocalyx. This aspect will be clarified in future research.

Furthermore, the well-organized intestinal epithelial cells are firmly held together by tight junctions, which are formed by membrane-spanning proteins such as occludin and claudin, and adherens junctions, formed by the mutual binding of E-cadherin molecules on the cell lateral sides [46]. These cell–cell adhesion apparatuses create a barrier that prevents the passage of low-molecular-weight substances between intestinal epithelial cells, effectively hindering the influx of foreign substances into the intestinal tissue. Therefore, mice lacking claudins exhibit high paracellular permeability, leading to intestinal inflammation because of the leakage of luminal contents, including bacterial components, and increased paracellular organic solute flux [47–49].

## Immunoglobulin A

IgA is most abundant among immunoglobulins in the gut, which is constitutively secreted by intestinal plasma cells differentiated from IgM^+^ B cells in gut associated lymphoid tissue (GALT) such as Peyer’s patches and isolated lymphoid follicles in the lamina propria. Dimeric IgA from plasma cells in the lamina propria is transported to the luminal side across intestinal epithelial cells via polymeric immunoglobulin receptor (pIgR) and released into the intestinal lumen as secretory IgA (SIgA). The role of SIgA in the gut mucosal barrier system is divided into defending against pathogenic microorganisms and maintaining the composition of symbiotic bacteria. One of the primary functions of SIgA is to neutralize pathogens and toxins in the gut lumen. By binding to bacteria, viruses, or toxins, SIgA prevents them from adhering to and penetrating the epithelial cells of the gut, thereby preventing infection without driving an excessive inflammatory response. SIgA not only prevents infections but also preferentially coats pathogenic commensal bacteria with polyreactive binding, maintaining a robust balance of microbial species through preventing the overgrowth of inflammatory commensals [50–52]. Therefore, IgA-deficiency causes microbial dysbiosis in human although IgM and IgG compensate immunoglobulin coating of bacteria [53, 54].

## Regulation of the mucosal barrier system by recognizing gut bacterial signals

**Fig. 2:**
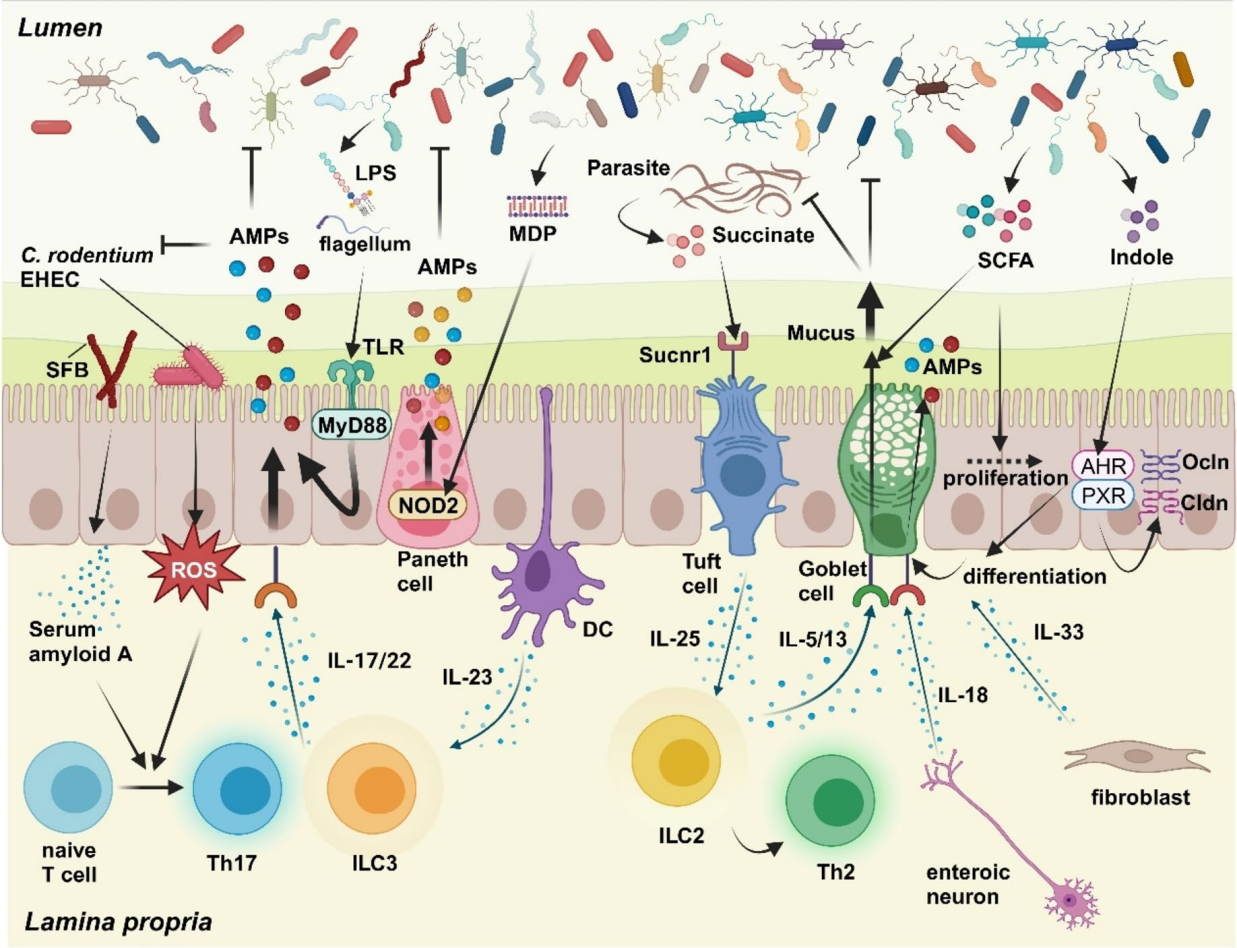
Regulation of the mucosal barrier system in the gut by intestinal microorganisms and lamina propria cells. Intestinal epithelial cells sense signals from commensal bacteria and pathogenic microorganisms in the lumen and cytokines from various lamina propria cells. This sensing mechanism allows for the dynamic regulation of the mucosal barrier system to adapt to changes in the gut environment. Intestinal epithelial cells express pattern recognition receptors, such as Toll-like receptors (TLRs) and Nucleotide-binding oligomerization domain-containing protein 2 (NOD2), which detect bacterial components like lipopolysaccharides (LPS), flagellum, and muramyl dipeptide (MDP). Activation of these receptors leads to the production of antimicrobial peptides (AMPs). Attachment of segmented filamentous bacteria (SFB) and pathogenic bacteria such as *Citrobacter rodentium* and enterohemorrhagic *Escherichia coli* (EHEC) triggers the production of serum amyloid A and reactive oxygen species (ROS), respectively. These promote the differentiation of T helper 17 (Th17) cells, resulting in the secretion of IL-17 and IL-22, which, in turn, enhance AMP production by intestinal epithelial cells. Furthermore, IL-22 produced by type3 innate lymphoid cells (ILC3), activated by IL-23 from dendritic cells (DC) in response to commensal bacteria, enhances the production of AMPs. Tuft cells, sensing succinate produced by parasites, produce IL-25, leading to the activation of type2 innate lymphoid cells (ILC2) and Th2 cells. IL-5 and IL-13 from these activated cells stimulate goblet cells to produce and secrete mucins, aiding in parasite elimination. Short chain fatty acids (SCFAs) from commensal bacteria promote mucus production by goblet cells and the proliferation of intestinal epithelial cells. Indole, a tryptophan metabolite, stimulates the differentiation of goblet cells and the expression of tight-junction molecules, such as Occludin (Ocln) and Claudin (Cldn), through the activation of Aryl hydrocarbon receptor (AHR) and pregnane X receptor (PXR). Additionally, enteric neurons release IL-18, promoting AMP production by goblet cells, while fibroblasts release IL-33, which supports goblet cell differentiation. These processes highlight the intricate regulation of the mucosal barrier by various bacterial signals and cytokines, crucial for maintaining gut homeostasis in response to environmental changes

The gut microbial composition is diverse among individuals and easily changes due to diet, stress, and many other environmental factors. To regulate various gut microbiota and its change, intestinal epithelial cells, which directly encounter intestinal bacteria, can sense signals from symbiotic or invading pathogenic bacteria (Fig. 2). Depending on the signals they receive, these cells strengthen the mucosal barrier and transmit signals to immune cells located in the lamina propria. This contribution is significant for adapting to changes in the intestinal environment, such as alterations in the intestinal microbiota and infections caused by pathogenic bacteria. Signals from intestinal bacteria to intestinal epithelial cells can be classified into three types: bacterial metabolites, bacterial components, and bacterial adhesion.

## Bacterial metabolites

Representative metabolites produced by intestinal bacteria include short-chain fatty acids (SCFAs), secondary bile acids and tryptophan metabolites. Several recent studies have elucidated their effects on intestinal epithelial cells.

SCFAs (e.g., butyrate, propionate and acetate) are generated by beneficial intestinal bacteria such as *Akkermansia muciniphila* and *Faecalibacterium prausnitziifrom* the breakdown of dietary fiber. SCFAs serve not only as an energy source for the host but also as modulators of the physiological functions of intestinal epithelial cells and immune cells [55]. In particular, butyrate plays a crucial role in maintaining intestinal epithelial homeostasis and promoting epithelial repair after mucosal injury by stimulating the proliferation of intestinal epithelial cells [56, 57]. Furthermore, butyrate activates peroxisome proliferator-activated receptor gamma (PPAR-γ) signaling in colonic intestinal epithelial cells [58]. This activation inhibits the expansion of Enterobacteriaceae bacteria by reducing the bioavailability of respiratory electron acceptors [58]. SCFAs also activate the MUC2 gene promoter in goblet cells, leading to increased mucus production and release from goblet cells [59–63]. As for SCFAs from pathogens, succinate, an SCFA produced by invading helminths, is recognized by tuft cells, a type of epithelial cell bearing brush-like microvilli, via Succinate receptor 1 (Sucnr1). Activated tuft cells, in turn, produce interleukin (IL)-25, which activates type 2 innate lymphoid cells (ILC2) [64]. IL-13, produced by activated ILC2, triggers a Th2 response, contributing to helminth elimination by enhancing mucus production by goblet cells [65, 66].

Some cholic acid (CA) and chenodeoxycholic acid (CDCA) that are not reabsorbed in the intestinal tract are converted by intestinal bacteria into secondary bile acids [67]. CA is converted to deoxycholic acid and CDCA is converted to ursodeoxycholic acid and lithocholic acid [67]. Ursodeoxycholic acid promotes wound healing by enhancing the migration of intestinal epithelial cells to villi via the epidermal growth factor receptor/cyclooxygenase-2 pathway [68]. Additionally, intestinal epithelial endocrine cells have been found to recognize secondary bile acids through the Takeda G protein-coupled receptor 5 (TGR5) [69]. Deoxycholic acid, for example, stimulates TGR5 in enteroendocrine cells, promoting colon peristalsis through the release of 5-hydroxytryptamine (5-HT) and calcitonin gene-related peptide (CGRP), with both acting as major transmitters in the afferent limb of the peristaltic reflex [69].

Tryptophan, an amino acid found in food, is converted to indole in the intestinal tract by tryptophanase, an enzyme produced by intestinal bacteria. Intestinal epithelial cells express the aryl hydrocarbon receptor (AHR) and pregnane X receptor (PXR), which are receptors that recognize indole [70–72]. AHR deficiency in intestinal epithelial cells has been linked to increased susceptibility to pathogenic bacterial infection because of reduced expression of Muc2 and carbonic anhydrase 4 (Car4) [73]. Additionally, indole stimulates the expression of cell junction-associated molecules, including occludin and claudins, through PXR activation [72, 74]. These findings indicate that the activation of AHR and PXR in intestinal epithelial cells by indole reinforces the barrier function of intestinal epithelial cells.

## Bacterial components

Intestinal epithelial cells express several pattern recognition receptors, such as Toll-like receptors (TLRs) and NOD-like receptors (NLRs), for immune surveillance. These receptors enable the detection of bacterial components such as LPS, flagellin and peptidoglycans from the gut microbiota, initiating signal transduction pathways that lead to epithelial cell proliferation, cytokine production, antimicrobial molecule release and mucus production. In the small intestine, TLR/myeloid differentiation factor (MyD)88 signaling plays a pivotal role in the production of antimicrobial molecules by intestinal epithelial cells, particularly by Paneth cells [15, 75]. MyD88 deficiency in epithelial cells reduces the production of antimicrobial molecules in small intestinal epithelial cells, resulting in increased bacterial colonization on the intestinal epithelial surface and reduced resistance to infections caused by pathogenic bacteria such as *S*. Typhimurium [15, 76]. In the colon, TLR2, TLR4 and TLR5 are expressed at both the apical and basal sites of intestinal epithelial cells [77]. Mice lacking MyD88 in intestinal epithelial cells exhibit reduced AMP and mucus production in colonic intestinal epithelial cells, rendering them highly susceptible to an experimental colitis model and receptive to pathogenic bacterial infection [76, 78]. Cytoplasmic receptors, such as NLRs, also contribute to the maintenance of mucosal barrier function. Nucleotide-binding oligomerization domain-containing protein 2 (NOD2), an NLR, is highly expressed in Paneth cells and recognizes the muramyl dipeptide, a conserved structure in bacterial peptidoglycans. This recognition leads to enhanced production of AMPs [79–81].

## Bacterial adhesion

As mentioned earlier, the intestinal mucosal surface is shielded by well-organized epithelial barriers. Nevertheless, certain enteric and pathogenic bacteria can breach this barrier and adhere to the epithelial cell surface. Bacterial adhesion induces specific gene expression in epithelial cells. SFB, unique to rodents like mice or rats, inhabit the intestinal tract by adhering to epithelial cells in the ileum. Adhesion of SFB to epithelial cells triggers the production of serum amyloid A (SAA) by epithelial cells, a factor that strongly promotes the differentiation of T helper (Th) 17 cells, a subset of helper T cells found in the lamina propria [82, 83]. Th17 and ILC3 cells produce interleukin-17 (IL-17) and interleukin-22 (IL-22), which stimulate the secretion of antimicrobial molecules, including the Reg3 family of proteins, from intestinal epithelial cells, contributing to intestinal homeostasis [84, 85]. Furthermore, the adhesion of *C. rodentium*, a murine pathogenic bacterium, to the colonic epithelium is associated with producing reactive oxygen species (ROS) in the epithelial cells. This ROS production promotes the differentiation of Th17 cells in the colonic lamina propria [82]. In addition, a recent paper demonstrated that major histocompatibility complex (MHC) class II of distal colonic epithelial cells is required for elimination of pathogenic bacteria such as *C. rodentium* through eliciting sustained IL-22 signaling from *C. rodentium*-specific T cells [86].

## Regulation of the mucosal barrier system by cytokines from *lamina* propria cells

To adapt to alterations in the gut environment, not only epithelial cells but also immune cells and the other stromal cells in the lamina propria directly sense the bacterial signals and produce various cytokines to orchestrate the mucosal barrier system (Fig. 2). Therefore, intestinal epithelial cells express several receptors for cytokines to receive signals from immune cells. Among various cytokines produced by intestinal immune cells in the lamina propria, IL-23 and IL-22 cooperatively play a crucial role in maintaining the epithelial barrier [87–89]. IL-23, produced by myeloid cells such as CD103^+^CD11b^+^ dendritic cells and activated by specific bacteria like SFB, induces the secretion of IL-22 from ILC3 and Th17 cells [88, 90]. IL-22 binds to the IL-22 receptor, a heterodimer of IL-22R1 and IL-10RB, which is highly expressed by intestinal epithelial cells. This binding enhances the production of bactericidal molecules, including Reg3 family of proteins, tight junction proteins, and mucins in intestinal epithelial cells [91–93]. This production protects against the invasion of commensal and pathogenic bacteria into the intestinal tissue. Additionally, a previous study demonstrated that IL-22 is essential for epithelial repair and regeneration in the gut by promoting the proliferation of epithelial cells in cases of epithelial injury [94]. Regarding the similarities and differences of IL-22 functions on intestinal epithelial cells between the small and large intestines, a recent RNA-sequencing study using germ-free, *Il23-/-* and *Il22-/-. *mice revealed that the functions of the IL-23/IL-22 axis on intestinal epithelial cells are less dependent of gut microbiota in the large intestine, unlike the case in the small intestine [95]. Additionally, apart from the well-known functions of IL-22, this study highlighted the intestinal site-specific roles of the IL-23/IL-22 axis on epithelial cells, including the regulation of lipid absorption in the small intestine and the promotion of epithelial cell proliferation in the large intestine [95].

IL-17 is also a cytokine that plays a crucial role in maintaining the gut mucosal barrier function. IL-17 from immune cells such as Th17 and ILC3, activated by commensal bacteria, promotes the production of AMPs, including α-defensins, from intestinal epithelial cells. This contributes to regulating commensal bacteria and eliminating of pathogenic bacteria [84, 96]. IL-17 receptor (IL-17RA) signaling in epithelial cells also reportedly upregulates tight junction molecules such as occludin to limit excessive permeability and maintain epithelial integrity [97]. Moreover, a recent study demonstrated that IL-17RA signaling in intestinal stem cells induces the expression of atonal bHLH transcription factor 1 (ATOH1), promoting differentiation into secretory lineages such as Paneth, tuft, goblet, and enteroendocrine cells [98].

Other cytokines from Th cells are also critically involved in modulating stem cell renewal, differentiation and the maintenance of mucosal barrier functions. IL-10 from regulatory T cells was found to support stem cell renewal, maintaining the stem cell niche [99]. In addition, IL-10 reportedly upregulates the expression of tight junction molecules [100], and promotes mucus production in goblet cells by preventing protein misfolding and ER stress [101], contributing to the preservation of the mucus barrier. In contrast, Th2 cytokines such as IL-4, 5, and 13 promote mucus production from goblet cells, aiding the host’s defense against helminth infection [102, 103]. In this context, IL-25 from activated tuft cells enhances the production of Th2 cytokines from Th2 and ILC2 cells in the lamina propria [65, 66]. Conversely, interferon-gamma (IFN-γ) induces the extrusion and release of antimicrobial molecules from Paneth cells [104]. Additionally, a few papers reported that IFN-γ increases the permeability of gut epithelia by downregulating the expression of tight-junction molecules including Zonula occludens-1 and occuludin [105–107]. Moreover, several recent papers demonstrated that IFN-γ from activated T cells promotes cell death of intestinal epithelial cells in a caspase-3/7-dependent manner [108, 109]. These results suggest that IFN-γ functions more toward weakening the mucosal barrier.

Tumor necrosis factor (TNF)-α, an inflammatory cytokine like IFN-γ, also reportedly increases the permeability of gut epithelia by downregulating the expression of tight-junction molecules via nuclear factor-kappa B (NF-κB) activation, followed by myosin light chain kinase (MLCK) activation. Additionally, TNF-α induces caspase 3-dependent apoptosis of intestinal epithelial cells via TNF receptor 1 signaling. Overall, TNF-α disrupts the gut epithelial barrier by increasing epithelial permeability, inducing cell death, and impairing the intestinal barrier, leading to inflammatory states of the intestine.

Not only immune cells but also non-immune cells such as stromal cells and neurons were found to modulate epithelial integrity through cytokine production. IL-33 derived from fibroblasts around intestinal crypts reportedly shapes the differentiation of epithelial progenitors toward secretory lineages, including Paneth and goblet cells [110]. A recent report showed that IL-18 derived from enteric neurons induces AMP production from goblet cells to protect against infection by invasive bacteria [111]. Taken together, cytokines from lamina propria cells orchestrate mucosal barrier functions to regulate commensal bacteria and eliminate pathogenic microorganisms.

## Breakdown of gut homeostasis by mucosal barrier dysfunction

**Fig. 3:**
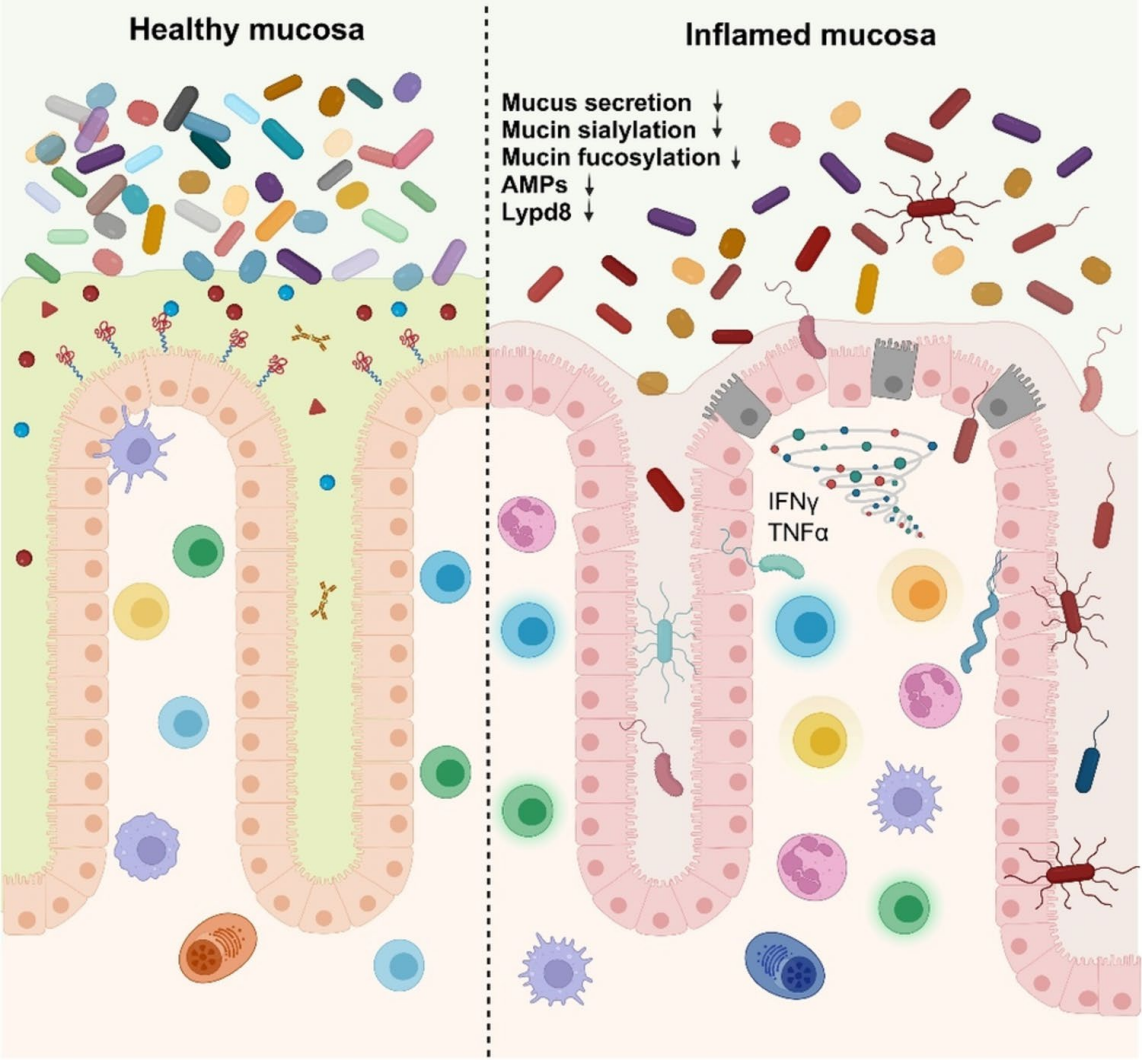
Intestinal inflammation because of mucosal barrier dysfunction. Under healthy conditions, the mucosal barrier system effectively segregates intestinal bacteria from intestinal epithelial cells. However, dysfunction of these mucosal barriers, characterized by reduced production of antimicrobial peptides (AMPs), mucus, Lypd8, and decreased fucosylation and sialylation of mucin, disrupts this segregation. This disruption leads to dysbiosis, allowing intestinal bacteria to breach the mucosal barrier and gain access to immune cells in the lamina propria. Consequently, activated immune cells respond by producing elevated levels of inflammatory cytokines, such as interferon-gamma (IFN-γ) and tumor necrosis factor-alpha (TNF-α). These cytokines, in turn, trigger the upregulation of cell death processes and increase the permeability of intestinal epithelial cells. As a result of these events, intestinal inflammation ensues, characterized by compromised barrier function, immune cell activation, and epithelial cell damage. This cascade of events underscores the critical role of the mucosal barrier system in preventing intestinal inflammation, and its dysfunction as a key factor in the development of such inflammation

When the intestinal epithelial cell function is impaired because of genetic factors or other environmental factors such as high-fat diet and drugs [112], the regulation of intestinal bacteria by the mucosal barrier is disrupted. This disruption facilitates an excessive host immune response to intestinal bacteria invading the epithelia and subsequent submucosa, leading to the development of chronic intestinal inflammation, as seen in IBD such as ulcerative colitis (UC) and Crohn's disease (CD) (Fig. 3) [113]. There is evidence for this, as the spontaneous onset of intestinal inflammation or increased susceptibility to intestinal inflammation has been reported in several genetically engineered mice with abnormal mucosal barriers.

The number of mucin-secreting goblet cells is well known to be decreased in the gut mucosa of both of UC and CD patients [114], suggesting that the reduced mucin production is a cause of accelerated intestinal inflammation in IBD. Indeed, in mice deficient in MUC2, the main component of intestinal mucus, a normal mucus layer is not formed, leading to intestinal bacteria invasion into the colonic mucosa and subsequent spontaneous onset of colitis [29, 115]. Additionally, a previous report demonstrated that *O*-glycosylation profile of MUC2 mucin is altered in UC patients and its alteration is associated with mucosal inflammation level, implying that *O*-glycosylation pattern of MUC2 mucin is involved in the pathology of IBD [116]. Deficiency of C1galt1, critical for core 1 *O*-glycosylation of mucin, results in the failure of normal mucus layer formation and spontaneous development of colitis, similar to MUC2-deficient mice [32]. Moreover, a defect in core 3 *O*-glycans in MUC2 also increases susceptibility to intestinal inflammation [31]. A recent paper showed that a missense mutation of *ST6GALNAC1* with loss of function of ST6GALNAC1, which mediates 2α-6 sialylation, was observed in UC patients [33]. St6galnac1-deficient mice were highly susceptible to DSS colitis because of the enhanced mucin degradation following dysbiosis [33]. Another recent study clearly demonstrated that mice devoid of St6galnac6, which is highly and selectively expressed in mouse colonic epithelia, develop spontaneous colitis because of the less lubricant mucus with the unidirectional Muc2 network structure due to the reduced sialylation [34]. Moreover, mice with a B3galt5 missense mutation, which was observed in IBD patients, showed mild spontaneous colitis because of the reduced sialylation of Muc2, similar to St6galnac6-deficient mice [34]. Concerning the other glycosyltransferases expressed in intestinal epithelial cells, fucosyltransferase 2 (FUT2) gene polymorphism was found to be associated with susceptibility to IBD in several genome-wide association studies [117–121]. Indeed. Fut2-deficient mice, in which α1,2 fucose is absent in the mucus, showed high susceptibility to DSS-induced colitis because of the increase in gut bacteria generating lysophosphatidylcholine, which damages the epithelial barrier [122]. These results indicate that the alteration of glycosylation in the mucus is involved in the pathogenesis of IBD.

A previous paper reported that LYPD8 expression is substantially reduced in the inflamed mucosa of UC patients [36]. This result was supported by a single cell RNA-sequencing analysis of intestinal epithelial cells from UC patients [123], suggesting that the reduced expression of LYPD8 is related to the progression of UC. Indeed, in mice devoid of Lypd8, flagellated bacteria such as *Proteus mirabilis*, which has been shown to be associated with intestinal inflammation [124], invade the colonic mucosa, resulting in high susceptibility to DSS-induced experimental intestinal inflammation and spontaneous onset of colitis in the case of a high-fat diet feeding [36, 125]. In addition, the treatment of recombinant LYPD8 protein alleviated intestinal inflammation in Lypd8-deficient mice [125].

Concerning the relationship between AMPs and the pathology of IBD, some previous reports indicate that the production of AMPs, including defensins and cathelicidin, is reduced in IBD patients, particularly those with CD patients [126, 127]. In some cases, this reduction has been associated with NOD2 mutations [128, 129]. Numerous animal studies have demonstrated that a compromised chemical barrier increases susceptibility to intestinal inflammation. Mice lacking either MyD88 in intestinal epithelia cells or systemic IL-22, which results in reduced production of AMPs from intestinal epithelia, are highly susceptible to DSS-induced intestinal inflammation [78, 130]. Mice deficient in *Nod2*, a gene identified as a disease susceptibility gene for IBD, were shown to be susceptible to CD-like intestinal inflammation upon infection with *Helicobacter hepaticus* because of the reduced production of AMPs from Paneth cells [80, 131, 132].

Furthermore, abnormalities in the mucosal barrier can cause dysbiosis, a disorder of the intestinal microbiota characterized by an increase in pathogenic bacteria and a reduction in bacterial diversity, which is observed in IBD patients. Dysbiosis has been reported to increases susceptibility to intestinal inflammation. For example, in mice deficient in NLR family Pyrin Domain Containing 6 (NLRP6), a member of the NLR family, mucus release from goblet cells is impaired, resulting in insufficient mucus layer formation [133, 134]. This impairment, in turn, causes dysbiosis and increased susceptibility to DSS-induced colitis [133, 134]. In recent years, dysbiosis has been shown to be associated with the onset and exacerbation of not only IBD but also autoimmune diseases such as rheumatoid arthritis, multiple sclerosis, and lifestyle-related diseases such as obesity and diabetes mellitus [135]. A recent study demonstrated a relationship between intestinal barrier dysfunction and the pathogenesis of a systemic disease. In this study, it was shown that the reduced production of AMPs because of a deficiency in the IL-23/IL-22 pathway induces the expansion of specific types of gut microbes producing trimethylamine N-oxide, exacerbating atherosclerosis induced by a high-fat diet [87]. Another paper showed that mice with high intestinal epithelial permeability develop more severe steatohepatitis after a high-fat diet than control mice [136]. These findings suggest that the mucosal barrier contributes not only to the prevention of intestinal inflammation by regulating intestinal bacteria but also to maintaining homeostasis of the immune and metabolic systems throughout the body.

## Conclusions and future directions

As described above, recent studies using various genetically engineered animals have revealed various barrier molecules produced by intestinal epithelial cells and their mechanisms of action. These studies have also revealed how the construction of barriers by these molecules is regulated by intestinal bacteria and immune cells. It is also becoming clear that deficiency or dysfunction of these barrier molecules contributes to the onset and development of IBD. However, there are still many unknowns regarding the dynamics of each barrier molecule, such as how individual bactericidal molecules bind to and kill bacteria in the intestinal lumen and how mucus flows to physically encompass bacteria. We believe improving labeling techniques for barrier molecules and intestinal bacteria and in-vivo imaging techniques for the intestinal lumen are necessary to elucidate these features of barrier molecules. In addition, an ex-vivo rheological assessment of mucus using atomic force microscopy, as shown in a recent paper, may also help in understanding the dynamics of mucus [34]. Moreover, using the recently developed mini-intestinal tube fabrication technology [137], we may be able to clarify the dynamics of barrier molecules and their regulatory mechanisms by aligning organoid-derived intestinal epithelial cells on a glass slide, seeding intestinal bacteria on the epithelium and immune cells and stromal cells under the epithelium, and observing their interaction with each other by live imaging. This approach may help elucidate the dynamics of barrier molecules and their regulatory mechanisms.

Differences in mucin glycosylation at intestinal sites have been highlighted at the molecular level in recent years [30, 34], however it remains unclear what function each glycosylation plays in each intestinal site. Furthermore, while glycosylation in the intestinal epithelia is reported to play an important role in the barrier function of molecules other than Muc2 and in the regulation of the intestinal microbiota, the precise role of glycosylation of molecules other than Muc2 remains unresolved. Although some of the details of mucin glycosylation in IBD patients have been clarified, the relationship between the pathophysiology of IBD and changes in mucin glycosylation also remains elusive. Therefore, it is expected that details about glycosylation in the mouse and human intestinal epithelia and the role of each glycosylation in the maintenance of intestinal homeostasis will be clarified by analyses of glycosyltransferase-mutant mice and glycomics of human intestinal samples from IBD patients. Further clarification of the intestinal mucosal barrier mechanism is expected to pave a new path to novel therapies for IBD by regulating the intestinal mucosal barrier system.

## Data Availability

Data sharing is not applicable to this article.

## References

[1] Wilde CG, Griffith JE, Marra MN, Snable JL, Scott RW (1989) Purification and characterization of human neutrophil peptide 4, a novel member of the defensin family. J Biol Chem 264:11200–11203 2500436

[2] Selsted ME, Harwig SS, Ganz T, Schilling JW, Lehrer RI (1985) Primary structures of three human neutrophil defensins. J Clin Invest 76:1436–1439. 10.1172/JCI112121 4056036 10.1172/JCI112121 PMC424095

[3] Brogden KA (2005) Antimicrobial peptides: pore formers or metabolic inhibitors in bacteria? Nat Rev Microbiol 3:238–250. 10.1038/nrmicro1098 15703760 10.1038/nrmicro1098

[4] Wilson CL, Ouellette AJ, Satchell DP, Ayabe T, Lopez-Boado YS, Stratman JL, Hultgren SJ, Matrisian LM, Parks WC (1999) Regulation of intestinal alpha-defensin activation by the metalloproteinase matrilysin in innate host defense. Science 286:113–117 10506557 10.1126/science.286.5437.113

[5] Selsted ME, Ouellette AJ (2005) Mammalian defensins in the antimicrobial immune response. Nat Immunol 6:551–557. 10.1038/ni1206 15908936 10.1038/ni1206

[6] Iimura M, Gallo RL, Hase K, Miyamoto Y, Eckmann L, Kagnoff MF (2005) Cathelicidin mediates innate intestinal defense against colonization with epithelial adherent bacterial pathogens. J Immunol 174:4901–4907 15814717 10.4049/jimmunol.174.8.4901

[7] Liang W, Enee E, Andre-Vallee C, Falcone M, Sun J, Diana J (2022) Intestinal Cathelicidin Antimicrobial Peptide Shapes a Protective Neonatal Gut Microbiota Against Pancreatic Autoimmunity. Gastroenterology 162(1288–1302):e1216. 10.1053/j.gastro.2021.12.272 10.1053/j.gastro.2021.12.272 34973295

[8] Memariani H, Memariani M (2023) Antibiofilm properties of cathelicidin LL-37: an in-depth review. World J Microbiol Biotechnol 39:99. 10.1007/s11274-023-03545-z 36781570 10.1007/s11274-023-03545-z

[9] Suzuki K, Murakami T, Kuwahara-Arai K, Tamura H, Hiramatsu K, Nagaoka I (2011) Human anti-microbial cathelicidin peptide LL-37 suppresses the LPS-induced apoptosis of endothelial cells. Int Immunol 23:185–193. 10.1093/intimm/dxq471 21393634 10.1093/intimm/dxq471

[10] Koziel J, Bryzek D, Sroka A, Maresz K, Glowczyk I, Bielecka E, Kantyka T, Pyrc K, Svoboda P, Pohl J, Potempa J (2014) Citrullination alters immunomodulatory function of LL-37 essential for prevention of endotoxin-induced sepsis. J Immunol 192:5363–5372. 10.4049/jimmunol.1303062 24771854 10.4049/jimmunol.1303062 PMC4036085

[11] Lehotzky RE, Partch CL, Mukherjee S, Cash HL, Goldman WE, Gardner KH, Hooper LV (2010) Molecular basis for peptidoglycan recognition by a bactericidal lectin. Proc Natl Acad Sci U S A 107:7722–7727. 10.1073/pnas.0909449107 20382864 10.1073/pnas.0909449107 PMC2867859

[12] Cash HL, Whitham CV, Behrendt CL, Hooper LV (2006) Symbiotic bacteria direct expression of an intestinal bactericidal lectin. Science 313:1126–1130. 10.1126/science.1127119 16931762 10.1126/science.1127119 PMC2716667

[13] Miki T, Holst O, Hardt WD (2012) The bactericidal activity of the C-type lectin RegIIIbeta against Gram-negative bacteria involves binding to lipid A. J Biol Chem 287:34844–34855. 10.1074/jbc.M112.399998 22896700 10.1074/jbc.M112.399998 PMC3464586

[14] Mukherjee S, Zheng H, Derebe MG, Callenberg KM, Partch CL, Rollins D, Propheter DC, Rizo J, Grabe M, Jiang QX, Hooper LV (2014) Antibacterial membrane attack by a pore-forming intestinal C-type lectin. Nature 505:103–107. 10.1038/nature12729nature12729[pii] 24256734 10.1038/nature12729 PMC4160023

[15] Vaishnava S, Yamamoto M, Severson KM, Ruhn KA, Yu X, Koren O, Ley R, Wakeland EK, Hooper LV (2011) The antibacterial lectin RegIIIgamma promotes the spatial segregation of microbiota and host in the intestine. Science 334:255–258. 10.1126/science.1209791 21998396 10.1126/science.1209791 PMC3321924

[16] Ragland SA, Criss AK (2017) From bacterial killing to immune modulation: Recent insights into the functions of lysozyme. PLoS Pathog 13:e1006512. 10.1371/journal.ppat.1006512 28934357 10.1371/journal.ppat.1006512 PMC5608400

[17] Dennis EA (1994) Diversity of group types, regulation, and function of phospholipase A2. J Biol Chem 269:13057–13060 8175726

[18] Rozenfeld RA, Liu X, DePlaen I, Hsueh W (2001) Role of gut flora on intestinal group II phospholipase A2 activity and intestinal injury in shock. Am J Physiol Gastrointest Liver Physiol 281:G957-963. 10.1152/ajpgi.2001.281.4.G957 11557516 10.1152/ajpgi.2001.281.4.G957

[19] Propheter DC, Chara AL, Harris TA, Ruhn KA, Hooper LV (2017) Resistin-like molecule beta is a bactericidal protein that promotes spatial segregation of the microbiota and the colonic epithelium. Proc Natl Acad Sci U S A 114:11027–11033. 10.1073/pnas.1711395114 28973871 10.1073/pnas.1711395114 PMC5651776

[20] Hogan SP, Seidu L, Blanchard C, Groschwitz K, Mishra A, Karow ML, Ahrens R, Artis D, Murphy AJ, Valenzuela DM, Yancopoulos GD, Rothenberg ME (2006) Resistin-like molecule beta regulates innate colonic function: barrier integrity and inflammation susceptibility. J Allergy Clin Immunol 118:257–268. 10.1016/j.jaci.2006.04.039 16815164 10.1016/j.jaci.2006.04.039 PMC1800427

[21] Hu Z, Zhang C, Sifuentes-Dominguez L, Zarek CM, Propheter DC, Kuang Z, Wang Y, Pendse M, Ruhn KA, Hassell B, Behrendt CL, Zhang B, Raj P, Harris-Tryon TA, Reese TA, Hooper LV (2021) Small proline-rich protein 2A is a gut bactericidal protein deployed during helminth infection. Science 374:eabe6723. 10.1126/science.abe6723 34735226 10.1126/science.abe6723 PMC8977106

[22] Hooper LV, Stappenbeck TS, Hong CV, Gordon JI (2003) Angiogenins: a new class of microbicidal proteins involved in innate immunity. Nat Immunol 4:269–273. 10.1038/ni888 12548285 10.1038/ni888

[23] Sultana MF, Suzuki M, Yamasaki F, Kubota W, Takahashi K, Abo H, Kawashima H (2022) Identification of Crucial Amino Acid Residues for Antimicrobial Activity of Angiogenin 4 and Its Modulation of Gut Microbiota in Mice. Front Microbiol 13:900948. 10.3389/fmicb.2022.900948 35733962 10.3389/fmicb.2022.900948 PMC9207454

[24] Sato T, van Es JH, Snippert HJ, Stange DE, Vries RG, van den Born M, Barker N, Shroyer NF, van de Wetering M, Clevers H (2011) Paneth cells constitute the niche for Lgr5 stem cells in intestinal crypts. Nature 469:415–418. 10.1038/nature09637 21113151 10.1038/nature09637 PMC3547360

[25] Stappenbeck TS, Hooper LV, Gordon JI (2002) Developmental regulation of intestinal angiogenesis by indigenous microbes via Paneth cells. Proc Natl Acad Sci U S A 99:15451–15455. 10.1073/pnas.202604299 12432102 10.1073/pnas.202604299 PMC137737

[26] Godl K, Johansson ME, Lidell ME, Morgelin M, Karlsson H, Olson FJ, Gum JR Jr, Kim YS, Hansson GC (2002) The N terminus of the MUC2 mucin forms trimers that are held together within a trypsin-resistant core fragment. J Biol Chem 277:47248–47256. 10.1074/jbc.M208483200 12374796 10.1074/jbc.M208483200

[27] Nilsson HE, Ambort D, Backstrom M, Thomsson E, Koeck PJB, Hansson GC, Hebert H (2014) Intestinal MUC2 mucin supramolecular topology by packing and release resting on D3 domain assembly. J Mol Biol 426:2567–2579. 10.1016/j.jmb.2014.04.027 24816392 10.1016/j.jmb.2014.04.027 PMC4293034

[28] Bergstrom KS, Xia L (2013) Mucin-type O-glycans and their roles in intestinal homeostasis. Glycobiology 23:1026–1037. 10.1093/glycob/cwt045 23752712 10.1093/glycob/cwt045 PMC3858029

[29] Johansson ME, Phillipson M, Petersson J, Velcich A, Holm L, Hansson GC (2008) The inner of the two Muc2 mucin-dependent mucus layers in colon is devoid of bacteria. Proc Natl Acad Sci U S A 105:15064–15069. 10.1073/pnas.0803124105 18806221 10.1073/pnas.0803124105 PMC2567493

[30] Bergstrom K, Shan X, Casero D, Batushansky A, Lagishetty V, Jacobs JP, Hoover C, Kondo Y, Shao B, Gao L, Zandberg W, Noyovitz B, McDaniel JM, Gibson DL, Pakpour S, Kazemian N, McGee S, Houchen CW, Rao CV, Griffin TM, Sonnenburg JL, McEver RP, Braun J, Xia L (2020) Proximal colon-derived O-glycosylated mucus encapsulates and modulates the microbiota. Science 370:467–472. 10.1126/science.aay7367 33093110 10.1126/science.aay7367 PMC8132455

[31] An G, Wei B, Xia B, McDaniel JM, Ju T, Cummings RD, Braun J, Xia L (2007) Increased susceptibility to colitis and colorectal tumors in mice lacking core 3-derived O-glycans. J Exp Med 204:1417–1429. 10.1084/jem.20061929 17517967 10.1084/jem.20061929 PMC2118614

[32] Fu J, Wei B, Wen T, Johansson ME, Liu X, Bradford E, Thomsson KA, McGee S, Mansour L, Tong M, McDaniel JM, Sferra TJ, Turner JR, Chen H, Hansson GC, Braun J, Xia L (2011) Loss of intestinal core 1-derived O-glycans causes spontaneous colitis in mice. J Clin Invest 121:1657–1666. 10.1172/JCI45538 21383503 10.1172/JCI45538 PMC3069788

[33] Yao Y, Kim G, Shafer S, Chen Z, Kubo S, Ji Y, Luo J, Yang W, Perner SP, Kanellopoulou C, Park AY, Jiang P, Li J, Baris S, Aydiner EK, Ertem D, Mulder DJ, Warner N, Griffiths AM, Topf-Olivestone C, Kori M, Werner L, Ouahed J, Field M, Liu C, Schwarz B, Bosio CM, Ganesan S, Song J, Urlaub H, Oellerich T, Malaker SA, Zheng L, Bertozzi CR, Zhang Y, Matthews H, Montgomery W, Shih HY, Jiang J, Jones M, Baras A, Shuldiner A, Gonzaga-Jauregui C, Snapper SB, Muise AM, Shouval DS, Ozen A, Pan KT, Wu C, Lenardo MJ (2022) Mucus sialylation determines intestinal host-commensal homeostasis. Cell 185(1172–1188):e1128. 10.1016/j.cell.2022.02.013 10.1016/j.cell.2022.02.013 PMC9088855 35303419

[34] Taniguchi M, Okumura R, Matsuzaki T, Nakatani A, Sakaki K, Okamoto S, Ishibashi A, Tani H, Horikiri M, Kobayashi N, Yoshikawa HY, Motooka D, Okuzaki D, Nakamura S, Kida T, Kameyama A, Takeda K (2023) Sialylation shapes mucus architecture inhibiting bacterial invasion in the colon. Mucosal Immunol. 10.1016/j.mucimm.2023.06.004 37385587 10.1016/j.mucimm.2023.06.004

[35] Arike L, Holmen-Larsson J, Hansson GC (2017) Intestinal Muc2 mucin O-glycosylation is affected by microbiota and regulated by differential expression of glycosyltranferases. Glycobiology 27:318–328. 10.1093/glycob/cww134 28122822 10.1093/glycob/cww134 PMC5444243

[36] Okumura R, Kurakawa T, Nakano T, Kayama H, Kinoshita M, Motooka D, Gotoh K, Kimura T, Kamiyama N, Kusu T, Ueda Y, Wu H, Iijima H, Barman S, Osawa H, Matsuno H, Nishimura J, Ohba Y, Nakamura S, Iida T, Yamamoto M, Umemoto E, Sano K, Takeda K (2016) Lypd8 promotes the segregation of flagellated microbiota and colonic epithelia. Nature 532:117–121. 10.1038/nature17406 27027293 10.1038/nature17406

[37] Hsu CC, Okumura R, Takeda K (2017) Human LYPD8 protein inhibits motility of flagellated bacteria. Inflamm Regen 37:23. 10.1186/s41232-017-0056-3 29259722 10.1186/s41232-017-0056-3 PMC5725809

[38] Okumura R, Kodama T, Hsu CC, Sahlgren BH, Hamano S, Kurakawa T, Iida T, Takeda K (2020) Lypd8 inhibits attachment of pathogenic bacteria to colonic epithelia. Mucosal Immunol 13:75–85. 10.1038/s41385-019-0219-4 31659301 10.1038/s41385-019-0219-4

[39] Ara T, Hashimoto D, Hayase E, Noizat C, Kikuchi R, Hasegawa Y, Matsuda K, Ono S, Matsuno Y, Ebata K, Ogasawara R, Takahashi S, Ohigashi H, Yokoyama E, Matsuo K, Sugita J, Onozawa M, Okumura R, Takeda K, Teshima T (2020) Intestinal goblet cells protect against GVHD after allogeneic stem cell transplantation via Lypd8. Sci Transl Med 12(550):eaaw0720. 10.1126/scitranslmed.aaw0720 32611682 10.1126/scitranslmed.aaw0720

[40] van Putten JPM, Strijbis K (2017) Transmembrane Mucins: Signaling Receptors at the Intersection of Inflammation and Cancer. J Innate Immun 9:281–299. 10.1159/000453594 28052300 10.1159/000453594 PMC5516414

[41] McGuckin MA, Every AL, Skene CD, Linden SK, Chionh YT, Swierczak A, McAuley J, Harbour S, Kaparakis M, Ferrero R, Sutton P (2007) Muc1 mucin limits both Helicobacter pylori colonization of the murine gastric mucosa and associated gastritis. Gastroenterology 133:1210–1218. 10.1053/j.gastro.2007.07.003 17919495 10.1053/j.gastro.2007.07.003

[42] McAuley JL, Linden SK, Png CW, King RM, Pennington HL, Gendler SJ, Florin TH, Hill GR, Korolik V, McGuckin MA (2007) MUC1 cell surface mucin is a critical element of the mucosal barrier to infection. J Clin Invest 117:2313–2324. 10.1172/JCI26705 17641781 10.1172/JCI26705 PMC1913485

[43] Linden SK, Sheng YH, Every AL, Miles KM, Skoog EC, Florin TH, Sutton P, McGuckin MA (2009) MUC1 limits Helicobacter pylori infection both by steric hindrance and by acting as a releasable decoy. PLoS Pathog 5:e1000617. 10.1371/journal.ppat.1000617 19816567 10.1371/journal.ppat.1000617 PMC2752161

[44] Zhou C, Liu Z, Liu Y, Fu W, Ding X, Liu J, Yu Y, Zhang Q (2013) Gene silencing of porcine MUC13 and ITGB5: candidate genes towards Escherichia coli F4ac adhesion. PLoS ONE 8:e70303. 10.1371/journal.pone.0070303 23922972 10.1371/journal.pone.0070303 PMC3726385

[45] Resta-Lenert S, Das S, Batra SK, Ho SB (2011) Muc17 protects intestinal epithelial cells from enteroinvasive E. coli infection by promoting epithelial barrier integrity. Am J Physiol Gastrointest Liver Physiol 300:G1144-1155. 10.1152/ajpgi.00138.2010 21393431 10.1152/ajpgi.00138.2010 PMC3119115

[46] Suzuki T (2013) Regulation of intestinal epithelial permeability by tight junctions. Cell Mol Life Sci 70:631–659. 10.1007/s00018-012-1070-x 22782113 10.1007/s00018-012-1070-x PMC11113843

[47] Tamura A, Kitano Y, Hata M, Katsuno T, Moriwaki K, Sasaki H, Hayashi H, Suzuki Y, Noda T, Furuse M, Tsukita S, Tsukita S (2008) Megaintestine in claudin-15-deficient mice. Gastroenterology 134:523–534. 10.1053/j.gastro.2007.11.040 18242218 10.1053/j.gastro.2007.11.040

[48] Muto S, Hata M, Taniguchi J, Tsuruoka S, Moriwaki K, Saitou M, Furuse K, Sasaki H, Fujimura A, Imai M, Kusano E, Tsukita S, Furuse M (2010) Claudin-2-deficient mice are defective in the leaky and cation-selective paracellular permeability properties of renal proximal tubules. Proc Natl Acad Sci U S A 107:8011–8016. 10.1073/pnas.0912901107 20385797 10.1073/pnas.0912901107 PMC2867900

[49] Ding L, Lu Z, Foreman O, Tatum R, Lu Q, Renegar R, Cao J, Chen YH (2012) Inflammation and disruption of the mucosal architecture in claudin-7-deficient mice. Gastroenterology 142:305–315. 10.1053/j.gastro.2011.10.025 22044670 10.1053/j.gastro.2011.10.025 PMC3267838

[50] Palm NW, de Zoete MR, Cullen TW, Barry NA, Stefanowski J, Hao L, Degnan PH, Hu J, Peter I, Zhang W, Ruggiero E, Cho JH, Goodman AL, Flavell RA (2014) Immunoglobulin A coating identifies colitogenic bacteria in inflammatory bowel disease. Cell 158:1000–1010. 10.1016/j.cell.2014.08.006 25171403 10.1016/j.cell.2014.08.006 PMC4174347

[51] Tang W, Wei Y, Ni Z, Hou K, Luo XM, Wang H (2024) IgA-mediated control of host-microbial interaction during weaning reaction influences gut inflammation. Gut Microbes 16:2323220. 10.1080/19490976.2024.2323220 38439579 10.1080/19490976.2024.2323220 PMC10936605

[52] Bunker JJ, Erickson SA, Flynn TM, Henry C, Koval JC, Meisel M, Jabri B, Antonopoulos DA, Wilson PC, Bendelac A (2017) Natural polyreactive IgA antibodies coat the intestinal microbiota. Science 358(6361):eaan6619. 10.1126/science.aan6619 28971969 10.1126/science.aan6619 PMC5790183

[53] Eriksen C, Moll JM, Myers PN, Pinto ARA, Danneskiold-Samsoe NB, Dehli RI, Rosholm LB, Dalgaard MD, Penders J, Jonkers DM, Pan-Hammarstrom Q, Hammarstrom L, Kristiansen K, Brix S (2023) IgG and IgM cooperate in coating of intestinal bacteria in IgA deficiency. Nat Commun 14:8124. 10.1038/s41467-023-44007-2 38065985 10.1038/s41467-023-44007-2 PMC10709418

[54] Catanzaro JR, Strauss JD, Bielecka A, Porto AF, Lobo FM, Urban A, Schofield WB, Palm NW (2019) IgA-deficient humans exhibit gut microbiota dysbiosis despite secretion of compensatory IgM. Sci Rep 9:13574. 10.1038/s41598-019-49923-2 31537840 10.1038/s41598-019-49923-2 PMC6753154

[55] Marchix J, Goddard G, Helmrath MA (2018) Host-Gut Microbiota Crosstalk in Intestinal Adaptation. Cell Mol Gastroenterol Hepatol 6:149–162. 10.1016/j.jcmgh.2018.01.024 30023411 10.1016/j.jcmgh.2018.01.024 PMC6047313

[56] Venkatraman A, Ramakrishna BS, Shaji RV, Kumar NS, Pulimood A, Patra S (2003) Amelioration of dextran sulfate colitis by butyrate: role of heat shock protein 70 and NF-kappaB. Am J Physiol Gastrointest Liver Physiol 285:G177-184. 10.1152/ajpgi.00307.2002 12637250 10.1152/ajpgi.00307.2002

[57] Ahmad MS, Krishnan S, Ramakrishna BS, Mathan M, Pulimood AB, Murthy SN (2000) Butyrate and glucose metabolism by colonocytes in experimental colitis in mice. Gut 46:493–499 10716678 10.1136/gut.46.4.493 PMC1727901

[58] Byndloss MX, Olsan EE, Rivera-Chavez F, Tiffany CR, Cevallos SA, Lokken KL, Torres TP, Byndloss AJ, Faber F, Gao Y, Litvak Y, Lopez CA, Xu G, Napoli E, Giulivi C, Tsolis RM, Revzin A, Lebrilla CB, Baumler AJ (2017) Microbiota-activated PPAR-gamma signaling inhibits dysbiotic Enterobacteriaceae expansion. Science 357:570–575. 10.1126/science.aam9949 28798125 10.1126/science.aam9949 PMC5642957

[59] Shimotoyodome A, Meguro S, Hase T, Tokimitsu I, Sakata T (2000) Short chain fatty acids but not lactate or succinate stimulate mucus release in the rat colon. Comp Biochem Physiol A Mol Integr Physiol 125:525–531 10840229 10.1016/s1095-6433(00)00183-5

[60] Finnie IA, Dwarakanath AD, Taylor BA, Rhodes JM (1995) Colonic mucin synthesis is increased by sodium butyrate. Gut 36:93–99 7890244 10.1136/gut.36.1.93 PMC1382360

[61] Barcelo A, Claustre J, Moro F, Chayvialle JA, Cuber JC, Plaisancie P (2000) Mucin secretion is modulated by luminal factors in the isolated vascularly perfused rat colon. Gut 46:218–224 10644316 10.1136/gut.46.2.218 PMC1727811

[62] Sakata T, Setoyama H (1995) Local stimulatory effect of short-chain fatty acids on the mucus release from the hindgut mucosa of rats (Rattus norvegicus). Comp Biochem Physiol A Physiol 111:429–432 7614035 10.1016/0300-9629(95)00033-4

[63] Burger-van Paassen N, Vincent A, Puiman PJ, van der Sluis M, Bouma J, Boehm G, van Goudoever JB, van Seuningen I, Renes IB (2009) The regulation of intestinal mucin MUC2 expression by short-chain fatty acids: implications for epithelial protection. Biochem J 420:211–219. 10.1042/BJ20082222 19228118 10.1042/BJ20082222

[64] Nadjsombati MS, McGinty JW, Lyons-Cohen MR, Jaffe JB, DiPeso L, Schneider C, Miller CN, Pollack JL, Nagana Gowda GA, Fontana MF, Erle DJ, Anderson MS, Locksley RM, Raftery D, von Moltke J (2018) Detection of Succinate by Intestinal Tuft Cells Triggers a Type 2 Innate Immune Circuit. Immunity 49(33–41):e37. 10.1016/j.immuni.2018.06.016 10.1016/j.immuni.2018.06.016 PMC6084797 30021144

[65] Gerbe F, Sidot E, Smyth DJ, Ohmoto M, Matsumoto I, Dardalhon V, Cesses P, Garnier L, Pouzolles M, Brulin B, Bruschi M, Harcus Y, Zimmermann VS, Taylor N, Maizels RM, Jay P (2016) Intestinal epithelial tuft cells initiate type 2 mucosal immunity to helminth parasites. Nature 529:226–230. 10.1038/nature16527 26762460 10.1038/nature16527 PMC7614903

[66] von Moltke J, Ji M, Liang HE, Locksley RM (2016) Tuft-cell-derived IL-25 regulates an intestinal ILC2-epithelial response circuit. Nature 529:221–225. 10.1038/nature16161 26675736 10.1038/nature16161 PMC4830391

[67] Hofmann AF, Hagey LR (2008) Bile acids: chemistry, pathochemistry, biology, pathobiology, and therapeutics. Cell Mol Life Sci 65:2461–2483. 10.1007/s00018-008-7568-6 18488143 10.1007/s00018-008-7568-6 PMC11131813

[68] Golden JM, Escobar OH, Nguyen MVL, Mallicote MU, Kavarian P, Frey MR, Gayer CP (2018) Ursodeoxycholic acid protects against intestinal barrier breakdown by promoting enterocyte migration via EGFR- and COX-2-dependent mechanisms. Am J Physiol Gastrointest Liver Physiol 315:G259–G271. 10.1152/ajpgi.00354.2017 29672156 10.1152/ajpgi.00354.2017 PMC6139640

[69] Alemi F, Poole DP, Chiu J, Schoonjans K, Cattaruzza F, Grider JR, Bunnett NW, Corvera CU (2013) The receptor TGR5 mediates the prokinetic actions of intestinal bile acids and is required for normal defecation in mice. Gastroenterology 144:145–154. 10.1053/j.gastro.2012.09.055 23041323 10.1053/j.gastro.2012.09.055 PMC6054127

[70] Hubbard TD, Murray IA, Bisson WH, Lahoti TS, Gowda K, Amin SG, Patterson AD, Perdew GH (2015) Adaptation of the human aryl hydrocarbon receptor to sense microbiota-derived indoles. Sci Rep 5:12689. 10.1038/srep12689 26235394 10.1038/srep12689 PMC4522678

[71] Hubbard TD, Murray IA, Perdew GH (2015) Indole and Tryptophan Metabolism: Endogenous and Dietary Routes to Ah Receptor Activation. Drug Metab Dispos 43:1522–1535. 10.1124/dmd.115.064246 26041783 10.1124/dmd.115.064246 PMC4576673

[72] Venkatesh M, Mukherjee S, Wang H, Li H, Sun K, Benechet AP, Qiu Z, Maher L, Redinbo MR, Phillips RS, Fleet JC, Kortagere S, Mukherjee P, Fasano A, Le Ven J, Nicholson JK, Dumas ME, Khanna KM, Mani S (2014) Symbiotic bacterial metabolites regulate gastrointestinal barrier function via the xenobiotic sensor PXR and Toll-like receptor 4. Immunity 41:296–310. 10.1016/j.immuni.2014.06.014 25065623 10.1016/j.immuni.2014.06.014 PMC4142105

[73] Metidji A, Omenetti S, Crotta S, Li Y, Nye E, Ross E, Li V, Maradana MR, Schiering C, Stockinger B (2018) The Environmental Sensor AHR Protects from Inflammatory Damage by Maintaining Intestinal Stem Cell Homeostasis and Barrier Integrity. Immunity 49(353–362):e355. 10.1016/j.immuni.2018.07.010 10.1016/j.immuni.2018.07.010 PMC6104739 30119997

[74] Shimada Y, Kinoshita M, Harada K, Mizutani M, Masahata K, Kayama H, Takeda K (2013) Commensal bacteria-dependent indole production enhances epithelial barrier function in the colon. PLoS ONE 8:e80604. 10.1371/journal.pone.0080604 24278294 10.1371/journal.pone.0080604 PMC3835565

[75] Ayabe T, Satchell DP, Wilson CL, Parks WC, Selsted ME, Ouellette AJ (2000) Secretion of microbicidal alpha-defensins by intestinal Paneth cells in response to bacteria. Nat Immunol 1:113–118. 10.1038/77783 11248802 10.1038/77783

[76] Bhinder G, Stahl M, Sham HP, Crowley SM, Morampudi V, Dalwadi U, Ma C, Jacobson K, Vallance BA (2014) Intestinal epithelium-specific MyD88 signaling impacts host susceptibility to infectious colitis by promoting protective goblet cell and antimicrobial responses. Infect Immun 82:3753–3763. 10.1128/IAI.02045-14 24958710 10.1128/IAI.02045-14 PMC4187802

[77] Price AE, Shamardani K, Lugo KA, Deguine J, Roberts AW, Lee BL, Barton GM (2018) A Map of Toll-like Receptor Expression in the Intestinal Epithelium Reveals Distinct Spatial, Cell Type-Specific, and Temporal Patterns. Immunity 49(560–575):e566. 10.1016/j.immuni.2018.07.016 10.1016/j.immuni.2018.07.016 PMC6152941 30170812

[78] Frantz AL, Rogier EW, Weber CR, Shen L, Cohen DA, Fenton LA, Bruno ME, Kaetzel CS (2012) Targeted deletion of MyD88 in intestinal epithelial cells results in compromised antibacterial immunity associated with downregulation of polymeric immunoglobulin receptor, mucin-2, and antibacterial peptides. Mucosal Immunol 5:501–512. 10.1038/mi.2012.23mi201223[pii] 22491177 10.1038/mi.2012.23 PMC3422608

[79] Hugot JP, Chamaillard M, Zouali H, Lesage S, Cezard JP, Belaiche J, Almer S, Tysk C, O’Morain CA, Gassull M, Binder V, Finkel Y, Cortot A, Modigliani R, Laurent-Puig P, Gower-Rousseau C, Macry J, Colombel JF, Sahbatou M, Thomas G (2001) Association of NOD2 leucine-rich repeat variants with susceptibility to Crohn’s disease. Nature 411:599–603. 10.1038/3507910735079107[pii] 11385576 10.1038/35079107

[80] Biswas A, Liu YJ, Hao L, Mizoguchi A, Salzman NH, Bevins CL, Kobayashi KS (2010) Induction and rescue of Nod2-dependent Th1-driven granulomatous inflammation of the ileum. Proc Natl Acad Sci U S A 107:14739–14744. 10.1073/pnas.1003363107 20679225 10.1073/pnas.1003363107 PMC2930434

[81] Ogura Y, Bonen DK, Inohara N, Nicolae DL, Chen FF, Ramos R, Britton H, Moran T, Karaliuskas R, Duerr RH, Achkar JP, Brant SR, Bayless TM, Kirschner BS, Hanauer SB, Nunez G, Cho JH (2001) A frameshift mutation in NOD2 associated with susceptibility to Crohn’s disease. Nature 411:603–606. 10.1038/35079114 11385577 10.1038/35079114

[82] Atarashi K, Tanoue T, Ando M, Kamada N, Nagano Y, Narushima S, Suda W, Imaoka A, Setoyama H, Nagamori T, Ishikawa E, Shima T, Hara T, Kado S, Jinnohara T, Ohno H, Kondo T, Toyooka K, Watanabe E, Yokoyama S, Tokoro S, Mori H, Noguchi Y, Morita H, Ivanov II, Sugiyama T, Nunez G, Camp JG, Hattori M, Umesaki Y, Honda K (2015) Th17 Cell Induction by Adhesion of Microbes to Intestinal Epithelial Cells. Cell 163:367–380. 10.1016/j.cell.2015.08.058S0092-8674(15)01110-1[pii] 26411289 10.1016/j.cell.2015.08.058 PMC4765954

[83] Ivanov II, Atarashi K, Manel N, Brodie EL, Shima T, Karaoz U, Wei D, Goldfarb KC, Santee CA, Lynch SV, Tanoue T, Imaoka A, Itoh K, Takeda K, Umesaki Y, Honda K, Littman DR (2009) Induction of intestinal Th17 cells by segmented filamentous bacteria. Cell 139:485–498. 10.1016/j.cell.2009.09.033S0092-8674(09)01248-3[pii] 19836068 10.1016/j.cell.2009.09.033 PMC2796826

[84] Liang SC, Tan XY, Luxenberg DP, Karim R, Dunussi-Joannopoulos K, Collins M, Fouser LA (2006) Interleukin (IL)-22 and IL-17 are coexpressed by Th17 cells and cooperatively enhance expression of antimicrobial peptides. J Exp Med 203:2271–2279. 10.1084/jem.20061308 16982811 10.1084/jem.20061308 PMC2118116

[85] Sano T, Huang W, Hall JA, Yang Y, Chen A, Gavzy SJ, Lee JY, Ziel JW, Miraldi ER, Domingos AI, Bonneau R, Littman DR (2015) An IL-23R/IL-22 Circuit Regulates Epithelial Serum Amyloid A to Promote Local Effector Th17 Responses. Cell 163:381–393. 10.1016/j.cell.2015.08.061S0092-8674(15)01113-7[pii] 26411290 10.1016/j.cell.2015.08.061 PMC4621768

[86] Zindl CL, Wilson CG, Chadha AS, Duck LW, Cai B, Harbour SN, Nagaoka-Kamata Y, Hatton RD, Gao M, Figge DA, Weaver CT (2024) Distal colonocytes targeted by C. rodentium recruit T-cell help for barrier defence. Nature 629:669–678. 10.1038/s41586-024-07288-1 38600382 10.1038/s41586-024-07288-1 PMC11096101

[87] Fatkhullina AR, Peshkova IO, Dzutsev A, Aghayev T, McCulloch JA, Thovarai V, Badger JH, Vats R, Sundd P, Tang HY, Kossenkov AV, Hazen SL, Trinchieri G, Grivennikov SI, Koltsova EK (2018) An Interleukin-23-Interleukin-22 Axis Regulates Intestinal Microbial Homeostasis to Protect from Diet-Induced Atherosclerosis. Immunity 49(943–957):e949. 10.1016/j.immuni.2018.09.011 10.1016/j.immuni.2018.09.011 PMC6257980 30389414

[88] Sano T, Huang W, Hall JA, Yang Y, Chen A, Gavzy SJ, Lee JY, Ziel JW, Miraldi ER, Domingos AI, Bonneau R, Littman DR (2016) An IL-23R/IL-22 Circuit Regulates Epithelial Serum Amyloid A to Promote Local Effector Th17 Responses. Cell 164:324. 10.1016/j.cell.2015.12.047 28915371 10.1016/j.cell.2015.12.047

[89] Keir M, Yi Y, Lu T, Ghilardi N (2020) The role of IL-22 in intestinal health and disease. J Exp Med 217:e20192195. 10.1084/jem.20192195 32997932 10.1084/jem.20192195 PMC7062536

[90] Kinnebrew MA, Buffie CG, Diehl GE, Zenewicz LA, Leiner I, Hohl TM, Flavell RA, Littman DR, Pamer EG (2012) Interleukin 23 production by intestinal CD103(+)CD11b(+) dendritic cells in response to bacterial flagellin enhances mucosal innate immune defense. Immunity 36:276–287. 10.1016/j.immuni.2011.12.011 22306017 10.1016/j.immuni.2011.12.011 PMC3288454

[91] Sugimoto K, Ogawa A, Mizoguchi E, Shimomura Y, Andoh A, Bhan AK, Blumberg RS, Xavier RJ, Mizoguchi A (2008) IL-22 ameliorates intestinal inflammation in a mouse model of ulcerative colitis. J Clin Invest 118:534–544. 10.1172/JCI33194 18172556 10.1172/JCI33194 PMC2157567

[92] Tsai PY, Zhang B, He WQ, Zha JM, Odenwald MA, Singh G, Tamura A, Shen L, Sailer A, Yeruva S, Kuo WT, Fu YX, Tsukita S, Turner JR (2017) IL-22 Upregulates Epithelial Claudin-2 to Drive Diarrhea and Enteric Pathogen Clearance. Cell Host Microbe 21(671–681):e674. 10.1016/j.chom.2017.05.009 10.1016/j.chom.2017.05.009 PMC5541253 28618266

[93] Zheng Y, Valdez PA, Danilenko DM, Hu Y, Sa SM, Gong Q, Abbas AR, Modrusan Z, Ghilardi N, de Sauvage FJ, Ouyang W (2008) Interleukin-22 mediates early host defense against attaching and effacing bacterial pathogens. Nat Med 14:282–289. 10.1038/nm1720 18264109 10.1038/nm1720

[94] Lindemans CA, Calafiore M, Mertelsmann AM, O’Connor MH, Dudakov JA, Jenq RR, Velardi E, Young LF, Smith OM, Lawrence G, Ivanov JA, Fu YY, Takashima S, Hua G, Martin ML, O’Rourke KP, Lo YH, Mokry M, Romera-Hernandez M, Cupedo T, Dow L, Nieuwenhuis EE, Shroyer NF, Liu C, Kolesnick R, van den Brink MRM, Hanash AM (2015) Interleukin-22 promotes intestinal-stem-cell-mediated epithelial regeneration. Nature 528:560–564. 10.1038/nature16460 26649819 10.1038/nature16460 PMC4720437

[95] Nakatani A, Okumura R, Ishibashi A, Okamoto S, Sakaki K, Ito Y, Okuzaki D, Inohara H, Takeda K (2023) Differential dependence on microbiota of IL-23/IL-22-dependent gene expression between the small- and large-intestinal epithelia. Genes Cells. 10.1111/gtc.13065 37680073 10.1111/gtc.13065

[96] Kumar P, Monin L, Castillo P, Elsegeiny W, Horne W, Eddens T, Vikram A, Good M, Schoenborn AA, Bibby K, Montelaro RC, Metzger DW, Gulati AS, Kolls JK (2016) Intestinal Interleukin-17 Receptor Signaling Mediates Reciprocal Control of the Gut Microbiota and Autoimmune Inflammation. Immunity 44:659–671. 10.1016/j.immuni.2016.02.007 26982366 10.1016/j.immuni.2016.02.007 PMC4794750

[97] Lee JS, Tato CM, Joyce-Shaikh B, Gulen MF, Cayatte C, Chen Y, Blumenschein WM, Judo M, Ayanoglu G, McClanahan TK, Li X, Cua DJ (2015) Interleukin-23-Independent IL-17 Production Regulates Intestinal Epithelial Permeability. Immunity 43:727–738. 10.1016/j.immuni.2015.09.003 26431948 10.1016/j.immuni.2015.09.003 PMC6044435

[98] Lin X, Gaudino SJ, Jang KK, Bahadur T, Singh A, Banerjee A, Beaupre M, Chu T, Wong HT, Kim CK, Kempen C, Axelrad J, Huang H, Khalid S, Shah V, Eskiocak O, Parks OB, Berisha A, McAleer JP, Good M, Hoshino M, Blumberg R, Bialkowska AB, Gaffen SL, Kolls JK, Yang VW, Beyaz S, Cadwell K, Kumar P (2022) IL-17RA-signaling in Lgr5(+) intestinal stem cells induces expression of transcription factor ATOH1 to promote secretory cell lineage commitment. Immunity 55(237–253):e238. 10.1016/j.immuni.2021.12.016 10.1016/j.immuni.2021.12.016 PMC8895883 35081371

[99] Biton M, Haber AL, Rogel N, Burgin G, Beyaz S, Schnell A, Ashenberg O, Su CW, Smillie C, Shekhar K, Chen Z, Wu C, Ordovas-Montanes J, Alvarez D, Herbst RH, Zhang M, Tirosh I, Dionne D, Nguyen LT, Xifaras ME, Shalek AK, von Andrian UH, Graham DB, Rozenblatt-Rosen O, Shi HN, Kuchroo V, Yilmaz OH, Regev A, Xavier RJ (2018) T Helper Cell Cytokines Modulate Intestinal Stem Cell Renewal and Differentiation. Cell 175(1307–1320):e1322. 10.1016/j.cell.2018.10.008 10.1016/j.cell.2018.10.008 PMC6239889 30392957

[100] Sun X, Yang H, Nose K, Nose S, Haxhija EQ, Koga H, Feng Y, Teitelbaum DH (2008) Decline in intestinal mucosal IL-10 expression and decreased intestinal barrier function in a mouse model of total parenteral nutrition. Am J Physiol Gastrointest Liver Physiol 294:G139-147. 10.1152/ajpgi.00386.2007 17991705 10.1152/ajpgi.00386.2007

[101] Hasnain SZ, Tauro S, Das I, Tong H, Chen AC, Jeffery PL, McDonald V, Florin TH, McGuckin MA (2013) IL-10 promotes production of intestinal mucus by suppressing protein misfolding and endoplasmic reticulum stress in goblet cells. Gastroenterology 144(357–368):e359. 10.1053/j.gastro.2012.10.043 10.1053/j.gastro.2012.10.043 23123183

[102] Else KJ, Finkelman FD, Maliszewski CR, Grencis RK (1994) Cytokine-mediated regulation of chronic intestinal helminth infection. J Exp Med 179:347–351. 10.1084/jem.179.1.347 8270879 10.1084/jem.179.1.347 PMC2191309

[103] Webb RA, Hoque T, Dimas S (2007) Expulsion of the gastrointestinal cestode, Hymenolepis diminuta by tolerant rats: evidence for mediation by a Th2 type immune enhanced goblet cell hyperplasia, increased mucin production and secretion. Parasite Immunol 29:11–21. 10.1111/j.1365-3024.2006.00908.x 17187651 10.1111/j.1365-3024.2006.00908.x

[104] Farin HF, Karthaus WR, Kujala P, Rakhshandehroo M, Schwank G, Vries RG, Kalkhoven E, Nieuwenhuis EE, Clevers H (2014) Paneth cell extrusion and release of antimicrobial products is directly controlled by immune cell-derived IFN-gamma. J Exp Med 211:1393–1405. 10.1084/jem.20130753 24980747 10.1084/jem.20130753 PMC4076587

[105] Youakim A, Ahdieh M (1999) Interferon-gamma decreases barrier function in T84 cells by reducing ZO-1 levels and disrupting apical actin. Am J Physiol 276:G1279-1288. 10.1152/ajpgi.1999.276.5.G1279 10330020 10.1152/ajpgi.1999.276.5.G1279

[106] Bruewer M, Utech M, Ivanov AI, Hopkins AM, Parkos CA, Nusrat A (2005) Interferon-gamma induces internalization of epithelial tight junction proteins via a macropinocytosis-like process. FASEB J 19:923–933. 10.1096/fj.04-3260com 15923402 10.1096/fj.04-3260com

[107] Smyth D, Phan V, Wang A, McKay DM (2011) Interferon-gamma-induced increases in intestinal epithelial macromolecular permeability requires the Src kinase Fyn. Lab Invest 91:764–777. 10.1038/labinvest.2010.208 21321534 10.1038/labinvest.2010.208

[108] Eriguchi Y, Nakamura K, Yokoi Y, Sugimoto R, Takahashi S, Hashimoto D, Teshima T, Ayabe T, Selsted ME, Ouellette AJ (2018) Essential role of IFN-gamma in T cell-associated intestinal inflammation. JCI Insight 3(18). 10.1172/jci.insight.121886 10.1172/jci.insight.121886 PMC6237234 30232288

[109] Yokoi T, Murakami M, Kihara T, Seno S, Arase M, Wing JB, Sondergaard JN, Kuwahara R, Minagawa T, Oguro-Igashira E, Motooka D, Okuzaki D, Mori R, Ikeda A, Sekido Y, Amano T, Iijima H, Ozono K, Mizushima T, Hirota S, Ikeuchi H, Takeda K (2023) Identification of a unique subset of tissue-resident memory CD4(+) T cells in Crohn’s disease. Proc Natl Acad Sci U S A 120:e2204269120. 10.1073/pnas.2204269120 36574662 10.1073/pnas.2204269120 PMC9910620

[110] Mahapatro M, Foersch S, Hefele M, He GW, Giner-Ventura E, McHedlidze T, Kindermann M, Vetrano S, Danese S, Gunther C, Neurath MF, Wirtz S, Becker C (2016) Programming of Intestinal Epithelial Differentiation by IL-33 Derived from Pericryptal Fibroblasts in Response to Systemic Infection. Cell Rep 15:1743–1756. 10.1016/j.celrep.2016.04.049 27184849 10.1016/j.celrep.2016.04.049

[111] Jarret A, Jackson R, Duizer C, Healy ME, Zhao J, Rone JM, Bielecki P, Sefik E, Roulis M, Rice T, Sivanathan KN, Zhou T, Solis AG, Honcharova-Biletska H, Velez K, Hartner S, Low JS, Qu R, de Zoete MR, Palm NW, Ring AM, Weber A, Moor AE, Kluger Y, Nowarski R, Flavell RA (2020) Enteric Nervous System-Derived IL-18 Orchestrates Mucosal Barrier Immunity. Cell 180:813–814. 10.1016/j.cell.2020.02.004 32084342 10.1016/j.cell.2020.02.004

[112] Chelakkot C, Ghim J, Ryu SH (2018) Mechanisms regulating intestinal barrier integrity and its pathological implications. Exp Mol Med 50:1–9. 10.1038/s12276-018-0126-x 30115904 10.1038/s12276-018-0126-x PMC6095905

[113] Okumura R, Takeda K (2017) Roles of intestinal epithelial cells in the maintenance of gut homeostasis. Exp Mol Med 49:e338. 10.1038/emm.2017.20 28546564 10.1038/emm.2017.20 PMC5454438

[114] Gersemann M, Becker S, Kubler I, Koslowski M, Wang G, Herrlinger KR, Griger J, Fritz P, Fellermann K, Schwab M, Wehkamp J, Stange EF (2009) Differences in goblet cell differentiation between Crohn’s disease and ulcerative colitis. Differentiation 77:84–94. 10.1016/j.diff.2008.09.008 19281767 10.1016/j.diff.2008.09.008

[115] Van der Sluis M, De Koning BA, De Bruijn AC, Velcich A, Meijerink JP, Van Goudoever JB, Buller HA, Dekker J, Van Seuningen I, Renes IB, Einerhand AW (2006) Muc2-deficient mice spontaneously develop colitis, indicating that MUC2 is critical for colonic protection. Gastroenterology 131:117–129. 10.1053/j.gastro.2006.04.020 16831596 10.1053/j.gastro.2006.04.020

[116] Larsson JM, Karlsson H, Crespo JG, Johansson ME, Eklund L, Sjovall H, Hansson GC (2011) Altered O-glycosylation profile of MUC2 mucin occurs in active ulcerative colitis and is associated with increased inflammation. Inflamm Bowel Dis 17:2299–2307. 10.1002/ibd.21625 21290483 10.1002/ibd.21625

[117] Franke A, McGovern DP, Barrett JC, Wang K, Radford-Smith GL, Ahmad T, Lees CW, Balschun T, Lee J, Roberts R, Anderson CA, Bis JC, Bumpstead S, Ellinghaus D, Festen EM, Georges M, Green T, Haritunians T, Jostins L, Latiano A, Mathew CG, Montgomery GW, Prescott NJ, Raychaudhuri S, Rotter JI, Schumm P, Sharma Y, Simms LA, Taylor KD, Whiteman D, Wijmenga C, Baldassano RN, Barclay M, Bayless TM, Brand S, Buning C, Cohen A, Colombel JF, Cottone M, Stronati L, Denson T, De Vos M, D’Inca R, Dubinsky M, Edwards C, Florin T, Franchimont D, Gearry R, Glas J, Van Gossum A, Guthery SL, Halfvarson J, Verspaget HW, Hugot JP, Karban A, Laukens D, Lawrance I, Lemann M, Levine A, Libioulle C, Louis E, Mowat C, Newman W, Panes J, Phillips A, Proctor DD, Regueiro M, Russell R, Rutgeerts P, Sanderson J, Sans M, Seibold F, Steinhart AH, Stokkers PC, Torkvist L, Kullak-Ublick G, Wilson D, Walters T, Targan SR, Brant SR, Rioux JD, D’Amato M, Weersma RK, Kugathasan S, Griffiths AM, Mansfield JC, Vermeire S, Duerr RH, Silverberg MS, Satsangi J, Schreiber S, Cho JH, Annese V, Hakonarson H, Daly MJ, Parkes M (2010) Genome-wide meta-analysis increases to 71 the number of confirmed Crohn’s disease susceptibility loci. Nat Genet 42:1118–1125. 10.1038/ng.717 21102463 10.1038/ng.717 PMC3299551

[118] McGovern DP, Jones MR, Taylor KD, Marciante K, Yan X, Dubinsky M, Ippoliti A, Vasiliauskas E, Berel D, Derkowski C, Dutridge D, Fleshner P, Shih DQ, Melmed G, Mengesha E, King L, Pressman S, Haritunians T, Guo X, Targan SR, Rotter JI, International IBDGC (2010) Fucosyltransferase 2 (FUT2) non-secretor status is associated with Crohn’s disease. Hum Mol Genet 19:3468–3476. 10.1093/hmg/ddq248 20570966 10.1093/hmg/ddq248 PMC2916706

[119] Aheman A, Luo HS, Gao F (2012) Association of fucosyltransferase 2 gene variants with ulcerative colitis in Han and Uyghur patients in China. World J Gastroenterol 18:4758–4764. 10.3748/wjg.v18.i34.4758 23002346 10.3748/wjg.v18.i34.4758 PMC3442215

[120] Parmar AS, Alakulppi N, Paavola-Sakki P, Kurppa K, Halme L, Farkkila M, Turunen U, Lappalainen M, Kontula K, Kaukinen K, Maki M, Lindfors K, Partanen J, Sistonen P, Matto J, Wacklin P, Saavalainen P, Einarsdottir E (2012) Association study of FUT2 (rs601338) with celiac disease and inflammatory bowel disease in the Finnish population. Tissue Antigens 80:488–493. 10.1111/tan.12016 23075394 10.1111/tan.12016

[121] Hu M, Zhang X, Li J, Chen L, He X, Sui T (2022) Fucosyltransferase 2: A Genetic Risk Factor for Intestinal Diseases. Front Microbiol 13:940196. 10.3389/fmicb.2022.940196 35923409 10.3389/fmicb.2022.940196 PMC9339987

[122] Tang X, Wang W, Hong G, Duan C, Zhu S, Tian Y, Han C, Qian W, Lin R, Hou X (2021) Gut microbiota-mediated lysophosphatidylcholine generation promotes colitis in intestinal epithelium-specific Fut2 deficiency. J Biomed Sci 28:20. 10.1186/s12929-021-00711-z 33722220 10.1186/s12929-021-00711-z PMC7958775

[123] Smillie CS, Biton M, Ordovas-Montanes J, Sullivan KM, Burgin G, Graham DB, Herbst RH, Rogel N, Slyper M, Waldman J, Sud M, Andrews E, Velonias G, Haber AL, Jagadeesh K, Vickovic S, Yao J, Stevens C, Dionne D, Nguyen LT, Villani AC, Hofree M, Creasey EA, Huang H, Rozenblatt-Rosen O, Garber JJ, Khalili H, Desch AN, Daly MJ, Ananthakrishnan AN, Shalek AK, Xavier RJ, Regev A (2019) Intra- and Inter-cellular Rewiring of the Human Colon during Ulcerative Colitis. Cell 178(714–730):e722. 10.1016/j.cell.2019.06.029 10.1016/j.cell.2019.06.029 PMC6662628 31348891

[124] Zhang J, Hoedt EC, Liu Q, Berendsen E, Teh JJ, Hamilton A, AW OB, Ching JYL, Wei H, Yang K, Xu Z, Wong SH, Mak JWY, Sung JJY, Morrison M, Yu J, Kamm MA, Ng SC (2021) Elucidation of Proteus mirabilis as a Key Bacterium in Crohn’s Disease Inflammation. Gastroenterology 160:317-330 e311. 10.1053/j.gastro.2020.09.036 33011176 10.1053/j.gastro.2020.09.036

[125] Hsu CC, Okumura R, Motooka D, Sasaki R, Nakamura S, Iida T, Takeda K (2021) Alleviation of colonic inflammation by Lypd8 in a mouse model of inflammatory bowel disease. Int Immunol 33:359–372. 10.1093/intimm/dxab012 33822948 10.1093/intimm/dxab012

[126] Nuding S, Fellermann K, Wehkamp J, Stange EF (2007) Reduced mucosal antimicrobial activity in Crohn’s disease of the colon. Gut 56:1240–1247. 10.1136/gut.2006.118646 17456510 10.1136/gut.2006.118646 PMC1954969

[127] Wehkamp J, Salzman NH, Porter E, Nuding S, Weichenthal M, Petras RE, Shen B, Schaeffeler E, Schwab M, Linzmeier R, Feathers RW, Chu H, Lima H Jr, Fellermann K, Ganz T, Stange EF, Bevins CL (2005) Reduced Paneth cell alpha-defensins in ileal Crohn’s disease. Proc Natl Acad Sci U S A 102:18129–18134. 10.1073/pnas.0505256102 16330776 10.1073/pnas.0505256102 PMC1306791

[128] Simms LA, Doecke JD, Walsh MD, Huang N, Fowler EV, Radford-Smith GL (2008) Reduced alpha-defensin expression is associated with inflammation and not NOD2 mutation status in ileal Crohn’s disease. Gut 57:903–910. 10.1136/gut.2007.142588 18305068 10.1136/gut.2007.142588

[129] Wehkamp J, Harder J, Weichenthal M, Schwab M, Schaffeler E, Schlee M, Herrlinger KR, Stallmach A, Noack F, Fritz P, Schroder JM, Bevins CL, Fellermann K, Stange EF (2004) NOD2 (CARD15) mutations in Crohn’s disease are associated with diminished mucosal alpha-defensin expression. Gut 53:1658–1664. 10.1136/gut.2003.032805 15479689 10.1136/gut.2003.032805 PMC1774270

[130] Zenewicz LA, Yancopoulos GD, Valenzuela DM, Murphy AJ, Stevens S, Flavell RA (2008) Innate and adaptive interleukin-22 protects mice from inflammatory bowel disease. Immunity 29:947–957. 10.1016/j.immuni.2008.11.003 19100701 10.1016/j.immuni.2008.11.003 PMC3269819

[131] Caruso R, Warner N, Inohara N, Nunez G (2014) NOD1 and NOD2: signaling, host defense, and inflammatory disease. Immunity 41:898–908. 10.1016/j.immuni.2014.12.010 25526305 10.1016/j.immuni.2014.12.010 PMC4272446

[132] Liu JZ, van Sommeren S, Huang H, Ng SC, Alberts R, Takahashi A, Ripke S, Lee JC, Jostins L, Shah T, Abedian S, Cheon JH, Cho J, Dayani NE, Franke L, Fuyuno Y, Hart A, Juyal RC, Juyal G, Kim WH, Morris AP, Poustchi H, Newman WG, Midha V, Orchard TR, Vahedi H, Sood A, Sung JY, Malekzadeh R, Westra HJ, Yamazaki K, Yang SK, International Multiple Sclerosis Genetics C, International IBDGC, Barrett JC, Alizadeh BZ, Parkes M, Bk T, Daly MJ, Kubo M, Anderson CA, Weersma RK (2015) Association analyses identify 38 susceptibility loci for inflammatory bowel disease and highlight shared genetic risk across populations. Nat Genet 47:979–986. 10.1038/ng.3359 26192919 10.1038/ng.3359 PMC4881818

[133] Elinav E, Strowig T, Kau AL, Henao-Mejia J, Thaiss CA, Booth CJ, Peaper DR, Bertin J, Eisenbarth SC, Gordon JI, Flavell RA (2011) NLRP6 inflammasome regulates colonic microbial ecology and risk for colitis. Cell 145:745–757. 10.1016/j.cell.2011.04.022S0092-8674(11)00480-6[pii] 21565393 10.1016/j.cell.2011.04.022 PMC3140910

[134] Wlodarska M, Thaiss CA, Nowarski R, Henao-Mejia J, Zhang JP, Brown EM, Frankel G, Levy M, Katz MN, Philbrick WM, Elinav E, Finlay BB, Flavell RA (2014) NLRP6 inflammasome orchestrates the colonic host-microbial interface by regulating goblet cell mucus secretion. Cell 156:1045–1059. 10.1016/j.cell.2014.01.026 24581500 10.1016/j.cell.2014.01.026 PMC4017640

[135] Miyauchi E, Shimokawa C, Steimle A, Desai MS, Ohno H (2023) The impact of the gut microbiome on extra-intestinal autoimmune diseases. Nat Rev Immunol 23:9–23. 10.1038/s41577-022-00727-y 35534624 10.1038/s41577-022-00727-y

[136] Rahman K, Desai C, Iyer SS, Thorn NE, Kumar P, Liu Y, Smith T, Neish AS, Li H, Tan S, Wu P, Liu X, Yu Y, Farris AB, Nusrat A, Parkos CA, Anania FA (2016) Loss of Junctional Adhesion Molecule A Promotes Severe Steatohepatitis in Mice on a Diet High in Saturated Fat, Fructose, and Cholesterol. Gastroenterology 151(733–746):e712. 10.1053/j.gastro.2016.06.022 10.1053/j.gastro.2016.06.022 PMC5037035 27342212

[137] Nikolaev M, Mitrofanova O, Broguiere N, Geraldo S, Dutta D, Tabata Y, Elci B, Brandenberg N, Kolotuev I, Gjorevski N, Clevers H, Lutolf MP (2020) Homeostatic mini-intestines through scaffold-guided organoid morphogenesis. Nature 585:574–578. 10.1038/s41586-020-2724-8 32939089 10.1038/s41586-020-2724-8

